# Core and Variable Fractions of the Rhizosphere Microbiome Across Five Field-Grown *Mentha* Accessions

**DOI:** 10.3390/plants15203081

**Published:** 2026-10-09

**Authors:** Yasmine Wazzani, Szilvia Tavaszi-Sárosi, Katalin Patonay, Ferenc Olasz, Ákos Juhász, Katalin Posta

**Affiliations:** 1 Agribiotechnology and Precision Breeding for Food Security National Laboratory, Institute of Genetics and Biotechnology, Hungarian University of Agriculture and Life Sciences, 2100 Gödöllő, Hungary; wazzani.yasmine@phd.uni-mate.hu (Y.W.); olasz.ferenc.gyorgy@uni-mate.hu (F.O.); posta.katalin@uni-mate.hu (K.P.); 2 Doctoral School of Natural Sciences, Hungarian University of Agriculture and Life Sciences, 2100 Gödöllő, Hungary; 3 Department of Medicinal and Aromatic Plants, Institute of Horticultural Science, Hungarian University of Agriculture and Life Sciences, 1118 Budapest, Hungary; tavaszi-sarosi.szilvia@uni-mate.hu; 4 Food and Wine Knowledge Centre, Eszterházy Károly Catholic University, 3300 Eger, Hungary; patonay.katalin@uni-eszterhazy.hu

**Keywords:** *Mentha*, rhizosphere, core microbiome, bacterial community, fungal community, amplicon sequencing

## Abstract

Rhizosphere microbiomes contain both conserved and variable community components, but the balance between these fractions may differ between bacteria and fungi. We compared bacterial and fungal rhizosphere communities associated with five field-grown *Mentha* accession plots (*Mentha spicata* B1, *Mentha* × *villosa* B10, *Mentha longifolia* B12, *Mentha* × *piperita* B20, and *Mentha suaveolens* J17) and managed inter-row soil using 16S rRNA gene and ITS amplicon sequencing, together with soil physicochemical characterization. Bacterial and fungal community composition differed significantly among the *Mentha* accession plots, while bacterial and fungal Bray–Curtis dissimilarities were moderately correlated. Because the five plant materials were represented by one field plot each, the observed between-group differences should be interpreted as plot-level patterns rather than replicated accession- or species-level effects. Prevalence-based analysis revealed a pronounced contrast between kingdoms: at the primary ≥2/3 within-plot prevalence threshold, 96.07% of bacterial amplicon sequence variants (ASVs) were shared across all five *Mentha* accession plots and accounted for 98.65% of bacterial reads, whereas only 21.89% of fungal ASVs were shared, although these still represented 72.83% of fungal reads. This contrast remained after rarefaction and under alternative prevalence thresholds. Soil physicochemical models were significant overall for both communities, but no individual soil variable showed a significant marginal effect. These results indicate that across the five sampled *Mentha* accession plots, bacterial communities were characterized by a highly shared ASV pool, whereas fungal communities showed substantially greater ASV-level variability.

## 1. Introduction

The rhizosphere represents a biologically active interface between plant roots and the surrounding soil, where microbial communities participate in nutrient transformations, plant nutrition, pathogen interactions, and responses to environmental condition and stress [1,2]. The composition of these communities is shaped by the interaction of environmental conditions with plant-associated factors, including host genotype, developmental stage, root architecture, and root-derived compounds [3,4,5,6]. Soil properties frequently represent major determinants of rhizosphere community structure, but plants can further modify the microbial assemblage recruited from the surrounding soil through differences in resource uptake, root morphology, and exudation patterns [4,5]. Consequently, the relative contributions of plant and environmental factors are context dependent, and even genetically related plant materials grown within a common environment may support differentiated rhizosphere communities [6].

Differences among rhizosphere microbial communities, however, represent only one aspect of plant-microbiome organization. Microbial taxa that occur consistently across individuals, genotypes, or environmental conditions can form a shared or core microbiome, whereas less consistently detected taxa constitute a more variable community fraction. The core microbiome concept has been applied using a wide range of prevalence and abundance criteria, and its estimated size therefore depends partly on the operational definition used [7,8]. Nevertheless, prevalence-based analyses can provide information that is not captured by diversity indices or ordination alone, because significant compositional differentiation may coexist with extensive taxonomic sharing. In wheat, for example, a core rhizosphere microbiome was maintained across contrasting genotypes, soils, agricultural systems, and geographic regions, with a relatively small set of consistently detected taxa accounting for a substantial proportion of community abundance [9]. Similarly, a geographically widespread core bacterial community represented a large fraction of root-associated reads in the medicinal plant *Anisodus tanguticus*, despite comprising only a small proportion of the total detected taxa [10]. Identifying both shared and variable fractions can therefore provide a complementary perspective on how plant-associated microbial communities are organized.

Bacterial and fungal communities may differ substantially in this balance between conserved and variable components. The two microbial kingdoms differ in dispersal capacity, lifestyle, resource use, environmental sensitivity, and responses to host-associated filtering. Large-scale sampling of the medicinal plant *Panax notoginseng* indicated stronger dispersal limitation and greater spatial heterogeneity in fungal than bacterial rhizosphere communities [11]. Host genetic variation can also affect the two kingdoms differently: in chrysanthemum, cultivar-related selection was more pronounced for bacterial communities, whereas fungal assembly showed a different and less directly genotype-linked pattern [12]. Such observations suggest that bacterial and fungal communities should not necessarily be expected to show equivalent degrees of taxonomic conservation, even when they occupy the same rhizosphere environment. Direct comparison of their shared and variable fractions may therefore reveal aspects of community organization that remain obscured when bacterial and fungal datasets are considered separately.

Species of *Mentha* are widely cultivated medicinal and aromatic plants characterized by considerable morphological and phytochemical diversity, particularly in their essential oil (EO) composition [13,14,15]. Despite their agricultural and commercial importance, direct information on *Mentha*-associated rhizosphere microbiota has historically been limited. Early culture-based work including *Mentha* × *piperita* demonstrated plant-associated differences in the abundance of several rhizosphere microbial groups among medicinal and aromatic species [16]. Culture-dependent work subsequently demonstrated a diverse population of fluorescent *Pseudomonas* in the *M.* × *piperita* rhizosphere and identified strains capable of promoting plant growth [17]. Community-level sequencing provided broader evidence that long-term cultivation of *Mentha arvensis* can alter bacterial and fungal diversity in both rhizosphere and bulk soil [18]. More recently, high-throughput 16S rRNA gene sequencing of several *Lamiaceae* species, including *M.* × *piperita*, demonstrated plant-associated differences in bacterial abundance and community structure relative to bulk soil [19].

Studies in other medicinal and aromatic *Lamiaceae* provide a broader context for these observations. Distinct fungal rhizosphere assemblages have been described among Mediterranean medicinal plants including *Origanum syriacum*, *Salvia fruticosa*, and *Teucrium capitatum*, with corresponding bacterial differentiation subsequently demonstrated in the same system [20,21]. Bacterial and fungal communities of *Salvia sclarea* changed during successive years of field cultivation, although the responses of the two kingdoms to mycorrhizal inoculation differed [22,23]. Developmental changes in rhizosphere microbial composition have also been observed in *Coleus barbatus* and *Salvia miltiorrhiza* [24,25], while fungal community structure varied with cultivation duration in lavender [26]. Culture-based studies have also isolated and characterized plant growth-promoting bacteria from the rhizosphere of *Minthostachys verticillata* [27]. In *Thymus* spp., bacterial assemblages from leaves, roots, and rhizosphere soil have been examined in relation to EO composition and tolerance [28]. More recently, combined rhizosphere sequencing and metabolite profiling has further demonstrated associations between microbial variation and plant biochemical characteristics in *Scutellaria baicalensis* [29]. Collectively, these studies show that medicinal and aromatic *Lamiaceae* can support structured and dynamic rhizosphere microbiota, with bacterial and fungal communities responding differently to plant identity, developmental stage, cultivation history, and local environmental conditions.

Recent work has begun to address these questions more directly within the genus *Mentha*. Previous studies comparing multiple *Mentha* species and field-grown *Mentha spicata* accession plots demonstrated substantial bacterial and fungal community structuring, together with extensive taxonomic overlap and associations with local soil conditions [30,31]. The extent to which microbial communities are reproducibly shared across multiple *Mentha* plant materials, the size and abundance of their variable fractions, and whether these patterns differ systematically between bacterial and fungal communities have not been the primary focus of previous *Mentha* studies.

The present study therefore examined the rhizosphere microbiomes associated with five field-grown *Mentha* accession plots (*M. spicata* B1, *M.* × *villosa* B10, *M. longifolia* B12, *M.* × *piperita* B20, and *M. suaveolens* J17) grown within the same experimental field under similar conditions and management. Our primary objective was to quantify the shared, core, and variable fractions of the bacterial and fungal communities using prevalence-based definitions and to determine whether their patterns of community conservation differed between the two microbial kingdoms. We additionally characterized community diversity and structure, bacterial–fungal compositional concordance, and associations with the measured soil physicochemical background, with managed inter-row soil included as a contextual reference; aerial-tissue EO profiles were used to document biochemical variation among the plant materials. We hypothesized that (i) a substantial microbial component would be reproducibly shared across the five *Mentha* accession plots and (ii) the relative balance between shared and variable community fractions would differ between bacterial and fungal communities.

## 2. Results

Comparisons among *Mentha* groups in the following sections are presented as contrasts among the five sampled accession plots, with the three samples within each plot representing spatial variation.

### 2.1. Soil Physicochemical Properties

All soil samples were classified as sandy loam, indicating broadly similar texture across the rhizosphere and managed inter-row soil samples. Despite this overall similarity, several chemical parameters differed significantly among the five sampled accession plots and managed inter-row soil (Table 1). Humus content differed significantly among groups (F_5,12_ = 15.19, *p* < 0.001, η^2^ = 0.864), with the highest mean value measured in the rhizosphere soil of *M.* × *villosa* B10 (2.46%) and the lowest in managed inter-row soil (1.86%). Relatively high humus contents were also recorded for *M. spicata* B1 and *M. longifolia* B12 (2.18% in both cases). Soil pH varied only within a narrow alkaline range (7.93–8.02), although the differences among groups were significant (F_5,12_ = 9.52, *p* < 0.001, η^2^ = 0.799); the highest mean pH was measured in managed inter-row soil (8.02). Nitrate + nitrite nitrogen showed the most pronounced variation (F_5,12_ = 125.12, *p* < 0.001, η^2^ = 0.981), reaching 17.27 mg/kg in the rhizosphere soil of *M. suaveolens* J17, while concentrations in the other groups were considerably lower, ranging from 1.64 mg/kg in *M. longifolia* B12 to 3.57 mg/kg in *M.* × *villosa* B10. Available sodium ranged from 41.70 mg/kg in *M. suaveolens* J17 to 56.00 mg/kg in *M.* × *piperita* B20, but the differences among groups did not reach statistical significance (F_5,12_ = 3.04, *p* = 0.053, η^2^ = 0.559). Available phosphorus also differed significantly (F_5,12_ = 5.01, *p* = 0.010, η^2^ = 0.676), with the highest mean concentration recorded for *M. spicata* B1 (740.67 mg/kg) and the lowest for *M.* × *piperita* B20 (582.33 mg/kg). Available potassium showed stronger differentiation among groups (F_5,12_ = 31.76, *p* < 0.001, η^2^ = 0.930): the highest concentration was measured in the rhizosphere soil of *M. spicata* B1 (226.00 mg/kg), whereas the lowest values occurred in *M. longifolia* B12 (83.80 mg/kg) and *M. suaveolens* J17 (88.77 mg/kg).

### 2.2. Essential Oil Composition

The EO composition of the five *Mentha* accessions is presented in Table S1. In total, 52 volatile compounds were identified. The main compound of *M. spicata* B1 and *M.* × *villosa* B10 was L-carvone (54.98% and 61.75%, respectively). Although both accessions were characterized by L-carvone-dominated oils, their other components differed considerably. *M. spicata* B1 contained relatively high proportions of trans-sabinene-hydrate (10.87%), 1,8-cineole (8.58%), and limonene (8.57%), whereas *M.* × *villosa* B10 was particularly rich in limonene (24.62%), with 1,8-cineole representing a further 5.54%. *M. longifolia* B12 showed a markedly different profile, dominated by cis-dihydrocarvone (39.98%) and trans-dihydrocarvone (29.17%); limonene (6.22%), germacrene-D (5.88%), and β-caryophyllene (5.01%) were also present in higher proportions. In *M.* × *piperita* B20, menthone (30.20%) and menthol (25.98%) were the main constituents, accompanied by isomenthone (6.66%), menthofuran (5.44%), and pulegone (5.41%). *M. suaveolens* J17 was characterized by a distinct cis-piperitone-epoxide dominated profile (64.43%), while γ-terpinene (8.92%) and piperitenone oxide (8.02%) were detected as the main other components.

### 2.3. Sequencing Output and Taxonomic Composition

After sequence processing and filtering, the bacterial 16S rRNA gene dataset comprised 2,410,664 sequences assigned to 3128 amplicon sequence variants (ASVs), while the fungal ITS dataset contained 1,882,583 sequences and 859 ASVs (Tables S2 and S3). Across individual samples, the bacterial libraries contained 112,125–167,262 sequences and 2590–3092 ASVs, whereas the fungal libraries contained 75,556–127,783 sequences and 238–508 ASVs. The sequencing and taxonomic summaries presented in this section are based on the processed, filtered, non-rarefied abundance tables.

**Figure 1:**
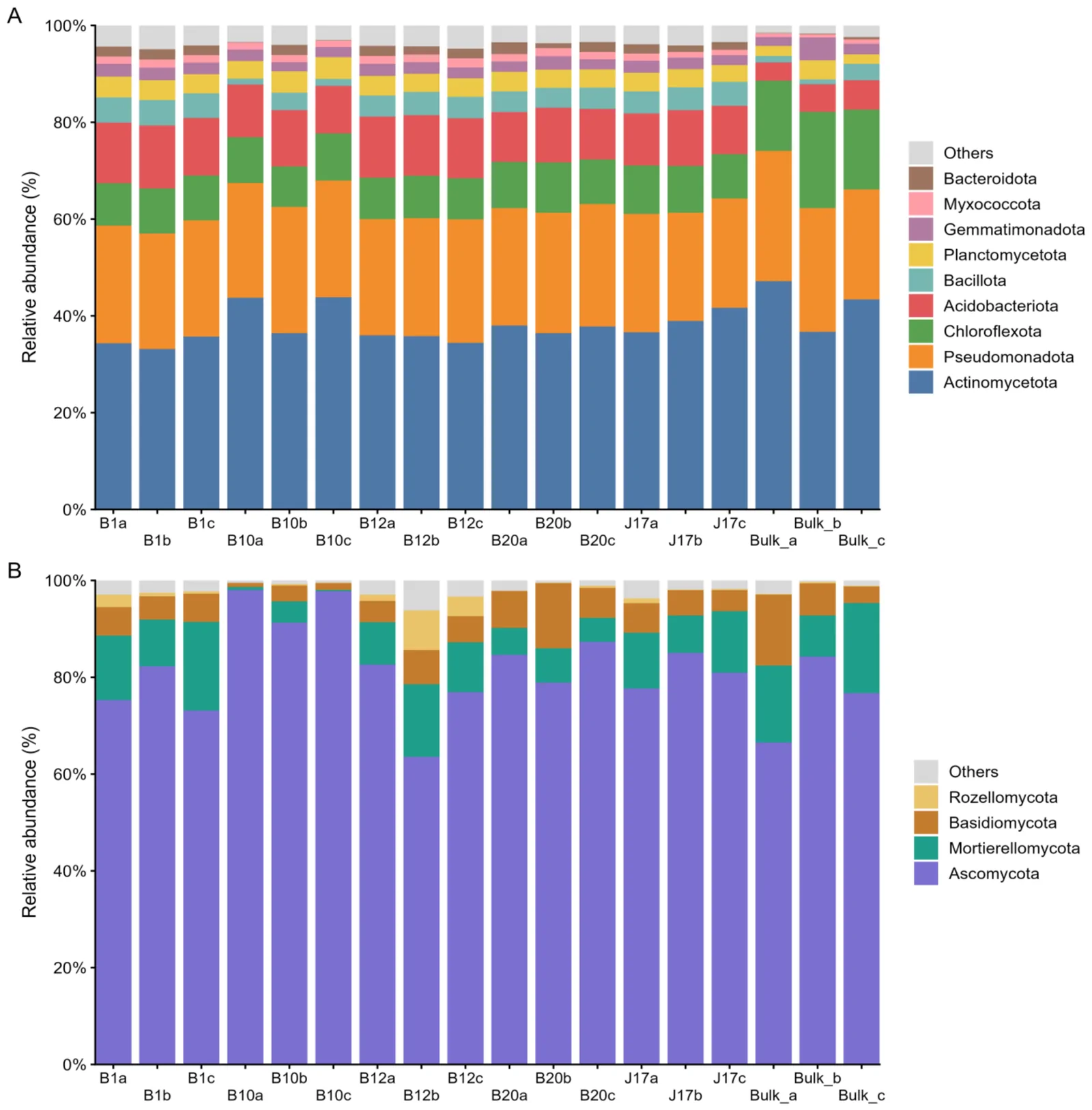
Phylum-level composition of bacterial (**A**) and fungal (**B**) communities in *Mentha* rhizosphere and managed inter-row soil samples. B1, *M. spicata*; B10, *M.* × *villosa*; B12, *M. longifolia*; B20, *M.* × *piperita*; J17, *M. suaveolens*. Within each *Mentha* accession, a, b, and c denote the three spatial soil samples collected from the same accession plot. Phyla with a mean relative abundance above 1% across the complete bacterial or fungal dataset are shown individually, while the remaining phyla are combined as “Others”.

A total of 27 bacterial phyla were detected (Table S2). Nine phyla had a mean relative abundance above 1% across the complete dataset and together accounted for 96.38% of the bacterial community; the remaining 18 phyla represented 3.62% (Figure 1A). The dominant phyla were Actinomycetota (38.34%), Pseudomonadota (24.39%), Chloroflexota (10.53%), Acidobacteriota (10.40%), Bacillota (3.79%), and Planctomycetota (3.73%). The overall phylum-level profiles were relatively similar among the five *Mentha* rhizosphere groups. Managed inter-row soil showed the clearest differences, particularly for Chloroflexota, which reached 16.98% compared with 8.63–9.70% in the rhizosphere soils, whereas Acidobacteriota accounted for only 5.19% in managed inter-row soil compared with 10.68–12.51% in the five *Mentha* rhizosphere groups. Bacillota and Bacteroidota were likewise less abundant in managed inter-row soil (1.90% and 0.24%, respectively) than in the rhizosphere samples.

Fungal composition was less evenly distributed among the samples (Figure 1B). Twelve fungal phyla were identified in total (Table S3), of which four exceeded 1% mean relative abundance and together represented 97.94% of the community; the remaining eight phyla accounted for 2.06%. Ascomycota was dominant overall (81.28%), followed by Mortierellomycota (9.62%), Basidiomycota (5.92%), and Rozellomycota (1.12%). The relative contribution of these phyla varied considerably among the sampled groups. *M.* × *villosa* B10 was strongly dominated by Ascomycota (95.69%), with Mortierellomycota and Basidiomycota accounting for only 1.78% and 1.83%, respectively. In contrast, Mortierellomycota reached 13.77% in *M. spicata* B1 and 14.32% in managed inter-row soil, while Basidiomycota represented 9.07% in *M.* × *piperita* B20 and 8.23% in managed inter-row soil. *M. longifolia* B12 had the highest mean proportion of Rozellomycota (4.50%).

### 2.4. Diversity of Bacterial and Fungal Communities

**Figure 2:**
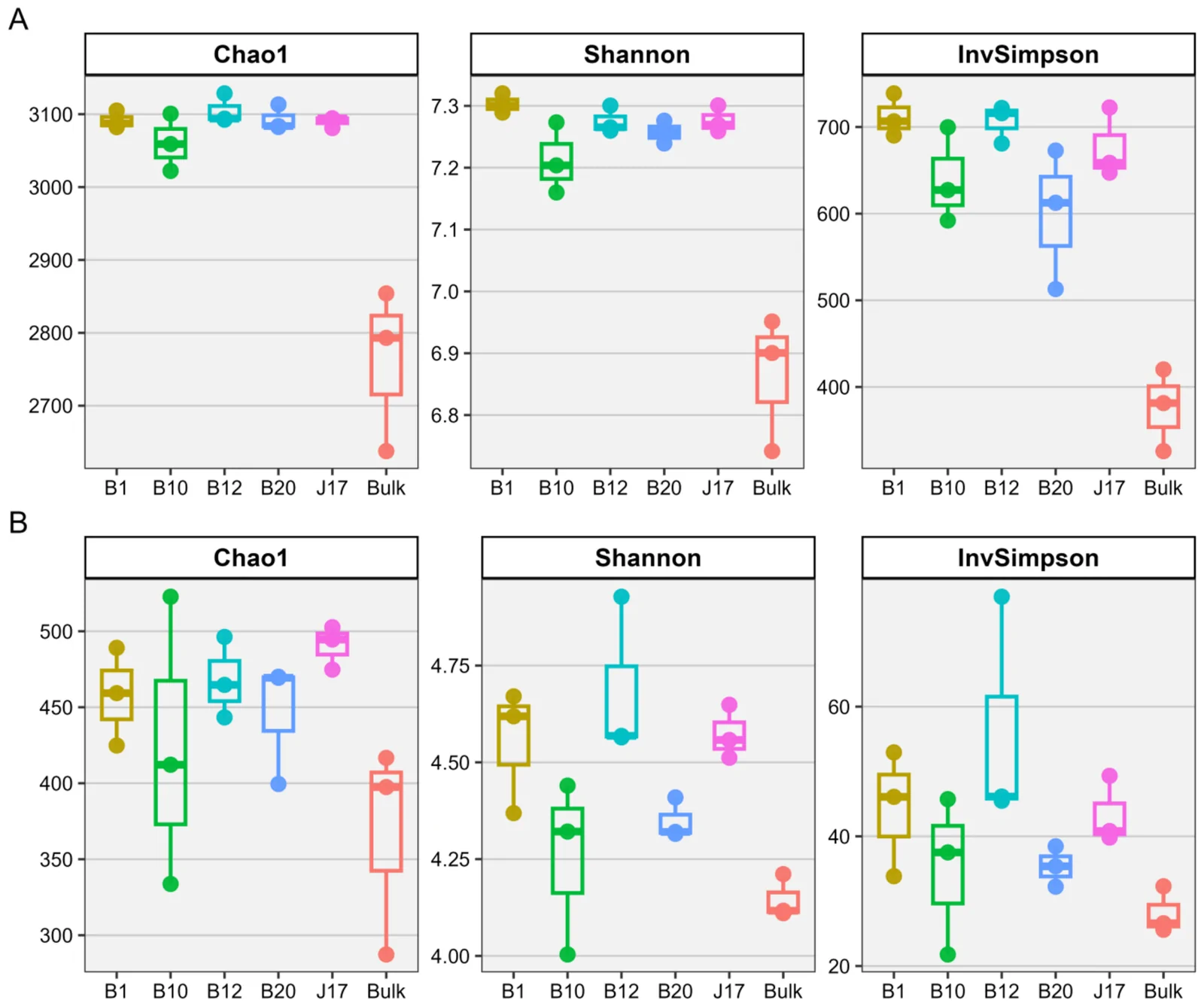
Alpha diversity of bacterial (**A**) and fungal (**B**) communities in *Mentha* rhizosphere and managed inter-row soil samples. B1, *M. spicata*; B10, *M.* × *villosa*; B12, *M. longifolia*; B20, *M.* × *piperita*; J17, *M. suaveolens*. Individual points represent the three spatial soil samples within each accession plot or the managed inter-row soil group. Statistical results, including Observed ASVs, are provided in Tables S4 and S5. Bulk denotes the managed inter-row soil samples.

For diversity analyses, the bacterial and fungal ASV abundance tables were rarefied to 112,125 and 75,556 sequences per sample, respectively. Alpha-diversity patterns were assessed using Chao1 richness, Shannon diversity, and inverse Simpson diversity (Figure 2), while Observed ASV richness and the complete statistical results are provided in Tables S4 and S5.

Bacterial alpha diversity differed significantly among the six sampled groups for all four indices (Observed ASVs: F_5,12_ = 31.61, *p* < 0.001; Chao1: F_5,12_ = 21.69, *p* < 0.001; Shannon: F_5,12_ = 29.69, *p* < 0.001; InvSimpson: F_5,12_ = 19.70, *p* < 0.001). These differences were primarily associated with managed inter-row soil, which had significantly lower values than all five *Mentha* rhizosphere groups. No significant pairwise differences were detected among the five sampled *Mentha* accession plots for any of the bacterial alpha-diversity indices.

Fungal alpha-diversity values showed greater within-group variability, particularly for richness estimates. Neither Observed ASV richness (F_5,12_ = 1.88, *p* = 0.171) nor Chao1 richness (F_5,12_ = 1.91, *p* = 0.166) differed significantly among groups, and the inverse Simpson index was not significant either (F_5,12_ = 2.79, *p* = 0.067). Shannon diversity, however, differed significantly among groups (F_5,12_ = 6.02, *p* = 0.005). The highest mean Shannon diversity was recorded for *M. longifolia* B12 (4.687), whereas managed inter-row soil had the lowest value (4.146). Tukey’s HSD indicated significantly lower Shannon diversity in managed inter-row soil than in *M. spicata* B1, *M. longifolia* B12, and *M. suaveolens* J17, while *M. longifolia* B12 also differed significantly from *M.* × *villosa* B10.

**Figure 3:**
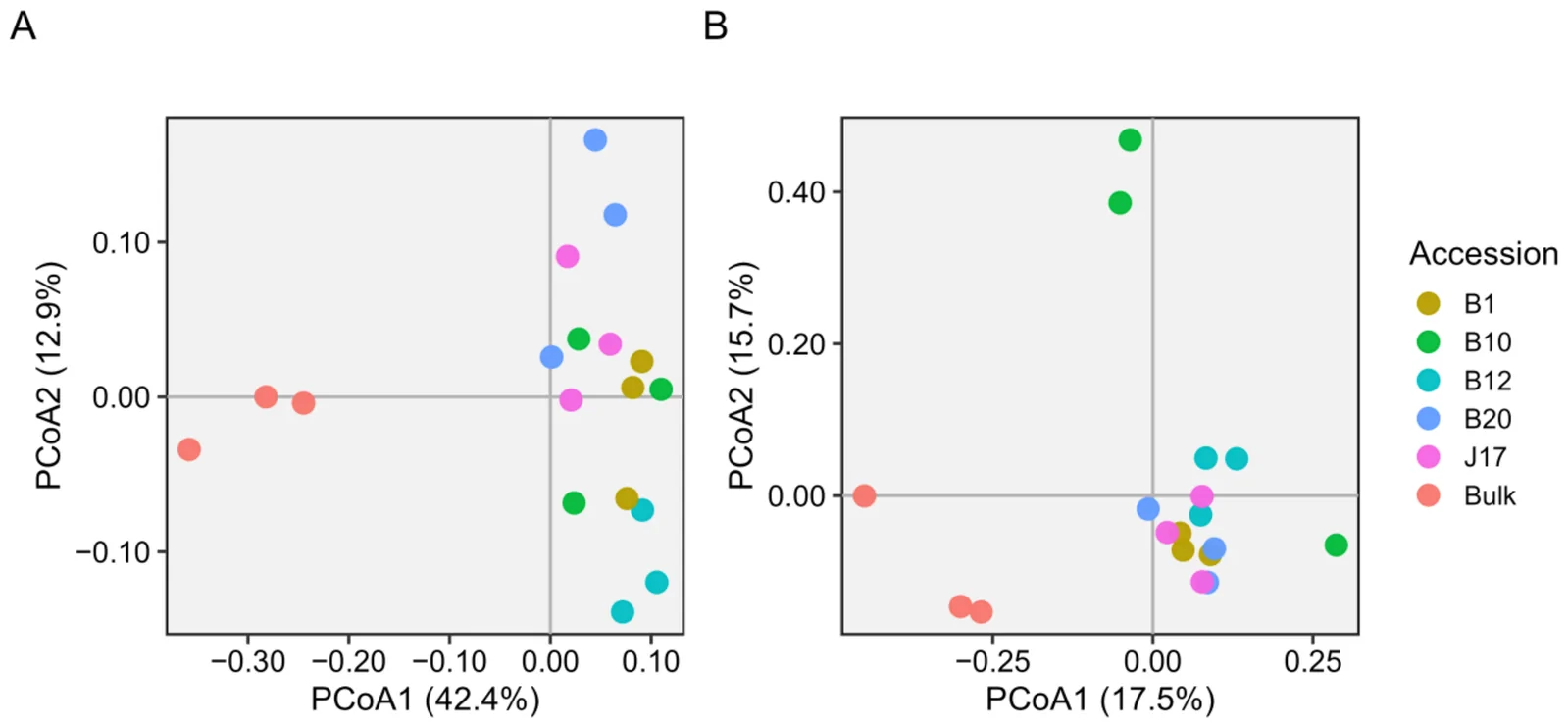
Bray–Curtis-based PCoA of bacterial (**A**) and fungal (**B**) communities in *Mentha* rhizosphere and managed inter-row soil samples. B1, *M. spicata*; B10, *M.* × *villosa*; B12, *M. longifolia*; B20, *M.* × *piperita*; J17, *M. suaveolens*. Each point represents one of the three spatial soil samples within an accession plot or managed inter-row soil group. Bulk denotes the managed inter-row soil samples.

Beta-diversity analysis based on Bray–Curtis dissimilarities showed clear structuring among the sampled groups (Figure 3A). The first two principal coordinate analysis (PCoA) axes explained 42.4% and 12.9% of the total variation, respectively, with managed inter-row soil separated from the *Mentha* rhizosphere samples and additional differentiation among the accession plots. Permutational multivariate analysis of variance (PERMANOVA) including all six groups detected significant differences in bacterial community composition (R^2^ = 0.641, F_5,12_ = 4.29, *p* < 0.001). Permutational analysis of multivariate dispersions (PERMDISP) also indicated significant heterogeneity in multivariate dispersion among groups (F_5,12_ = 6.37, *p* = 0.004). After excluding managed inter-row soil, differences among the five sampled *Mentha* accession plots remained significant (R^2^ = 0.528, F_4,10_ = 2.79, *p* < 0.001), while multivariate dispersion was no longer significant (F_4,10_ = 0.76, *p* = 0.574).

Fungal communities showed a less strongly structured ordination, with PCoA1 and PCoA2 explaining 17.5% and 15.7% of the variation, respectively (Figure 3B). PERMANOVA detected significant differences among all six groups (R^2^ = 0.512, F_5,12_ = 2.52, *p* < 0.001), while PERMDISP was not significant (F_5,12_ = 1.10, *p* = 0.401). Significant differences also remained among the five sampled *Mentha* accession plots after exclusion of managed inter-row soil (R^2^ = 0.454, F_4,10_ = 2.08, *p* < 0.001), with no significant difference in multivariate dispersion (F_4,10_ = 1.42, *p* = 0.277).

To assess whether bacterial and fungal community variation followed a similar pattern between samples, the corresponding Bray–Curtis dissimilarity matrices were compared using Mantel tests. A significant positive association was detected across all 18 samples (Spearman Mantel r = 0.419, *p* = 0.013), and a comparable relationship remained when the analysis was restricted to the 15 *Mentha* rhizosphere samples (r = 0.416, *p* = 0.002). Samples showing greater bacterial community dissimilarity also tended to exhibit greater fungal community dissimilarity, and this concordance was not driven solely by the rhizosphere-managed inter-row soil contrast.

### 2.5. Shared and Core Microbiome Across the Five Mentha Accession Plots

Core and shared-community analyses were performed using the filtered, non-rarefied ASV abundance tables. At the individual-sample level, 3062 of 3128 bacterial ASVs (97.89%) occurred in at least 12 of the 15 *Mentha* rhizosphere samples, compared with 243 of 859 fungal ASVs (28.29%) (Table 2). These ≥80% prevalence cores accounted for 99.33% of bacterial and 78.47% of fungal reads across the rhizosphere samples. Under the stricter criterion of detection in all 15 rhizosphere samples, 2762 bacterial ASVs (88.30%) and 93 fungal ASVs (10.83%) remained, representing 96.00% and 58.80% of bacterial and fungal reads, respectively. A universal core present in all 18 samples (15 rhizosphere and three managed inter-row soil samples) comprised 2158 bacterial ASVs (68.99%) but only 68 fungal ASVs (7.92%), accounting for 86.04% and 51.29% of rhizosphere reads, respectively.

**Figure 4:**
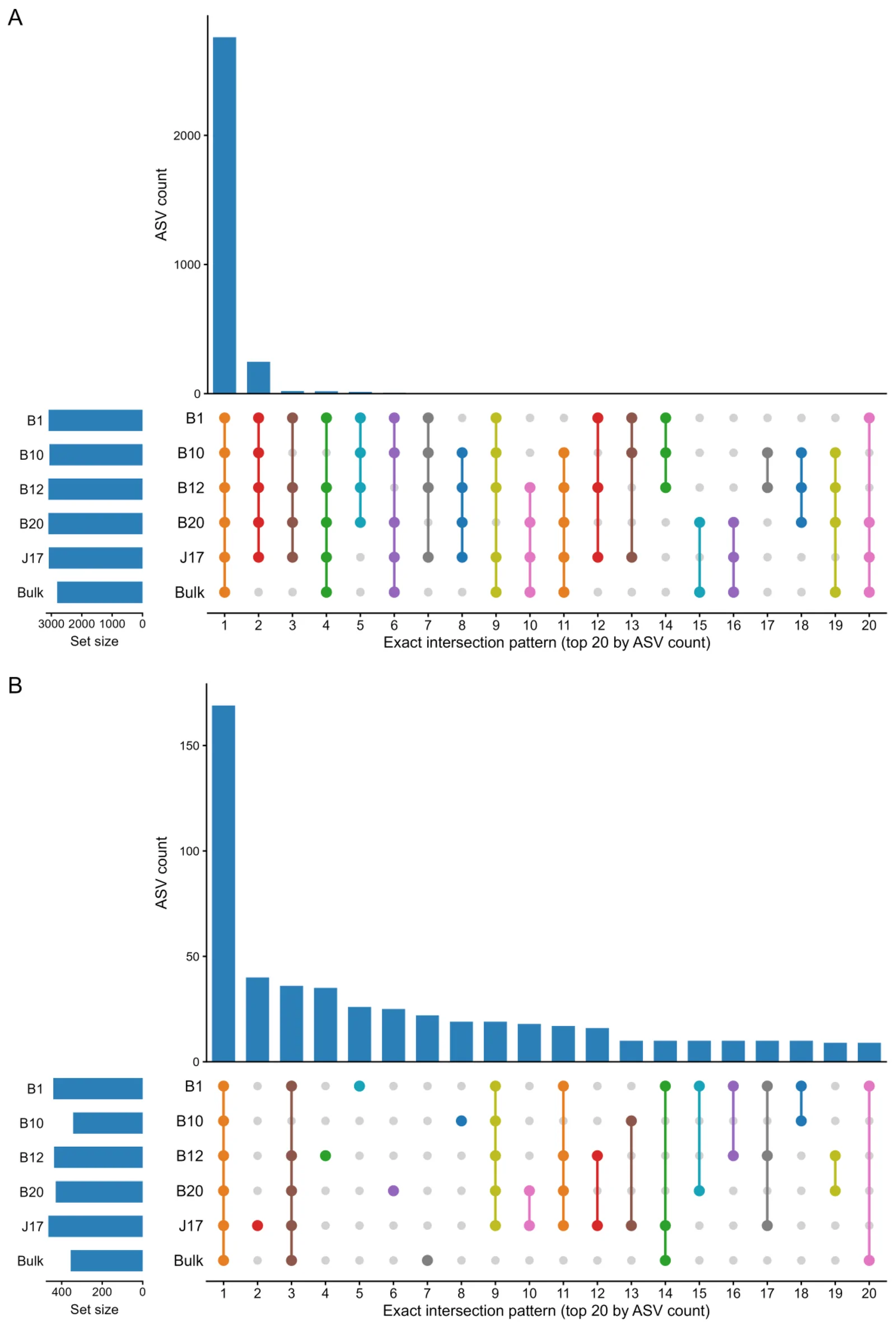
UpSet analysis of shared bacterial and fungal ASVs among five *Mentha* accession plots and managed inter-row soil. UpSet plots show exact intersections of bacterial 16S rRNA gene ASVs (**A**) and fungal ITS ASVs (**B**) among the five *Mentha* accession plots (B1, B10, B12, B20, and J17) and managed inter-row soil. Analyses were performed using the filtered, non-rarefied ASV count tables. An ASV was considered present in a given accession plot or managed inter-row soil group when it was detected (read count > 0) in at least two of the three spatial samples within that group (≥2/3 prevalence criterion). Horizontal bars on the left indicate the total number of ASVs satisfying this prevalence criterion within each group. Vertical bars indicate the number of ASVs belonging to each exact intersection pattern; the 20 largest intersections are shown. In the intersection matrices, colored dots identify the groups included in a given intersection and connecting lines link groups sharing the same exact ASV set, whereas pale grey dots indicate groups not included in that intersection. Intersection sizes are ordered from largest to smallest. Bulk denotes the managed inter-row soil samples.

Within-plot reproducibility across the five sampled *Mentha* accession plots also showed a large difference between the bacterial and fungal communities (Figure 4; Table 2). Using the primary ≥2/3 within-plot prevalence criterion, 3005 bacterial ASVs (96.07% of all bacterial ASVs) were detected in all five sampled accession plots, compared with 188 fungal ASVs (21.89%). Despite representing a much smaller proportion of the total fungal ASV pool, these shared fungal ASVs accounted for 72.83% of fungal reads, whereas the corresponding bacterial fraction represented 98.65% of bacterial reads. Of the ASVs shared across all five *Mentha* accession plots, 2759 of 3005 bacterial ASVs (91.81%) and 169 of 188 fungal ASVs (89.89%) also met the ≥2/3 prevalence criterion in managed inter-row soil. Using the sample-level ≥80% rhizosphere definition, 91 bacterial and 13 fungal ASVs were completely absent from managed inter-row soil; these rhizosphere-associated fractions represented 1.46% and 2.59% of bacterial and fungal reads, respectively (Table 2).

Sensitivity analyses using less and more stringent within-plot prevalence thresholds retained the same overall contrast between bacterial and fungal communities (Figures S1 and S2). At the ≥1/3 criterion, 3093 bacterial ASVs (98.88%) and 355 fungal ASVs (41.33%) were shared across all five *Mentha* accession plots, whereas requiring detection in all three samples of every accession plot reduced these numbers to 2762 bacterial ASVs (88.30%) and 93 fungal ASVs (10.83%). No bacterial ASV met the strict definition of being reproducibly associated with only one accession plot while absent from all other *Mentha* and managed inter-row soil samples. In contrast, 29 fungal ASVs fulfilled this criterion, comprising 3 ASVs in *M. spicata* B1, 3 in *M.* × *villosa* B10, 11 in *M. longifolia* B12, 7 in *M.* × *piperita* B20, and 5 in *M. suaveolens* J17; together, these accession-plot-associated exclusive fungal ASVs represented 0.79% of fungal reads.

Repeating the core and shared-community analyses with the rarefied abundance tables produced highly similar results (Table S6). At the primary ≥2/3 within-plot criterion, the fraction of ASVs shared across all five *Mentha* accession plots changed only from 96.07% to 95.40% for bacteria and from 21.89% to 20.61% for fungi. The corresponding cumulative abundances also remained very similar, indicating that the pronounced bacterial–fungal contrast was not materially affected by differences in sequencing depth.

To assess whether the bacterial–fungal contrast in shared-community size depended on ASV-level resolution, the analysis was repeated after taxonomic aggregation at genus and family ranks using the same ≥2/3 within-plot prevalence criterion (Table S7). In the fixed-rank analysis restricted to sequence clusters resolved to the respective taxonomic rank, 275 of 293 bacterial genera (93.86%) were shared across all five accession plots, compared with 75 of 234 fungal genera (32.05%). At family level, the corresponding proportions were 140 of 143 bacterial families (97.90%) and 48 of 130 fungal families (36.92%). Taxonomic resolution differed between the two marker datasets: 53.36% of bacterial and 16.38% of fungal rhizosphere reads were unresolved at genus level, while 25.55% and 6.44%, respectively, were unresolved at family level. Therefore, an additional sensitivity analysis retained unresolved lineages as separate operational taxonomic bins according to their existing lowest reliable taxonomic labels. Under this treatment, the shared proportions remained similar, at 95.38% and 31.67% for bacteria and fungi, respectively, after genus-column aggregation, and 98.03% and 37.25% after family-column aggregation. The corresponding shared operational bins accounted for 99.72% and 85.00% of bacterial and fungal rhizosphere reads at genus level and 99.93% and 92.22% at family level. Thus, taxonomic aggregation reduced the magnitude of the bacterial–fungal difference in feature sharing, particularly for fungi, but the contrast remained evident at both higher taxonomic ranks and under both treatments of unresolved assignments.

### 2.6. Taxonomic Identity of Core and Variable ASV Fractions

The taxonomic composition of the bacterial core and shared fractions was highly similar across the different prevalence definitions (Table S8). The strict rhizosphere core, the ≥80% prevalence core, and the shared fraction across the five sampled accession plots were dominated by members of Actinomycetota, Pseudomonadota, Acidobacteriota, and Chloroflexota. At lower taxonomic levels, recurrent abundant groups included unclassified Vicinamibacterales and Gaiellales, *Vicinamibacteraceae*, *Nocardioidaceae*, and the 67-14 lineage, while *Gaiella*, *Nocardioides*, and *Rubrobacter* were among the most abundant resolved genera. The much smaller rhizosphere-associated fraction showed a more heterogeneous composition, including *Chthoniobacteraceae*, *Pedosphaeraceae*, *Microscillaceae*, *Chitinophagaceae*, and *Sphingomonadaceae*; representative genera included *Chthoniobacter*, *Novosphingobium*, *Williamsia*, and *Chryseolinea*.

The fungal core was taxonomically more concentrated. The dominant families were consistent across the strict and ≥80% rhizosphere cores and the shared fraction identified across the five sampled accession plots, and included *Nectriaceae*, *Didymellaceae*, *Chaetomiaceae*, *Plectosphaerellaceae*, *Mortierellaceae*, and *Stachybotryaceae* (Table S8). Prominent genera included *Fusarium*, *Humicola*, *Plectosphaerella*, *Didymella*, *Tetracladium*, and *Striaticonidium*. The rhizosphere-associated fungal fraction contained only 13 ASVs and was represented mainly by *Plectosphaerellaceae*, *Bionectriaceae*, and Hypocreales-affiliated taxa, including *Paragibellulopsis*, *Nectriella*, *Fusariella*, *Beauveria*, and *Vishniacozyma*. The 29 accession-plot-associated exclusive fungal ASVs were distributed among all five *Mentha* accession plots and were taxonomically diverse, with no corresponding exclusive bacterial ASVs detected. These fungal ASVs included, among others, *Oliveonia* in *M. spicata* B1, *Preussia* in *M.* × *villosa* B10, Rozellomycota- and *Diversispora*-affiliated ASVs in *M. longifolia* B12, *Rhizophlyctis* and *Typhula* in *M.* × *piperita* B20, and *Ochronectria* and *Acrocalymma* in *M. suaveolens* J17.

### 2.7. Associations Between Soil Physicochemical Properties and Microbial Community Composition

**Figure 5:**
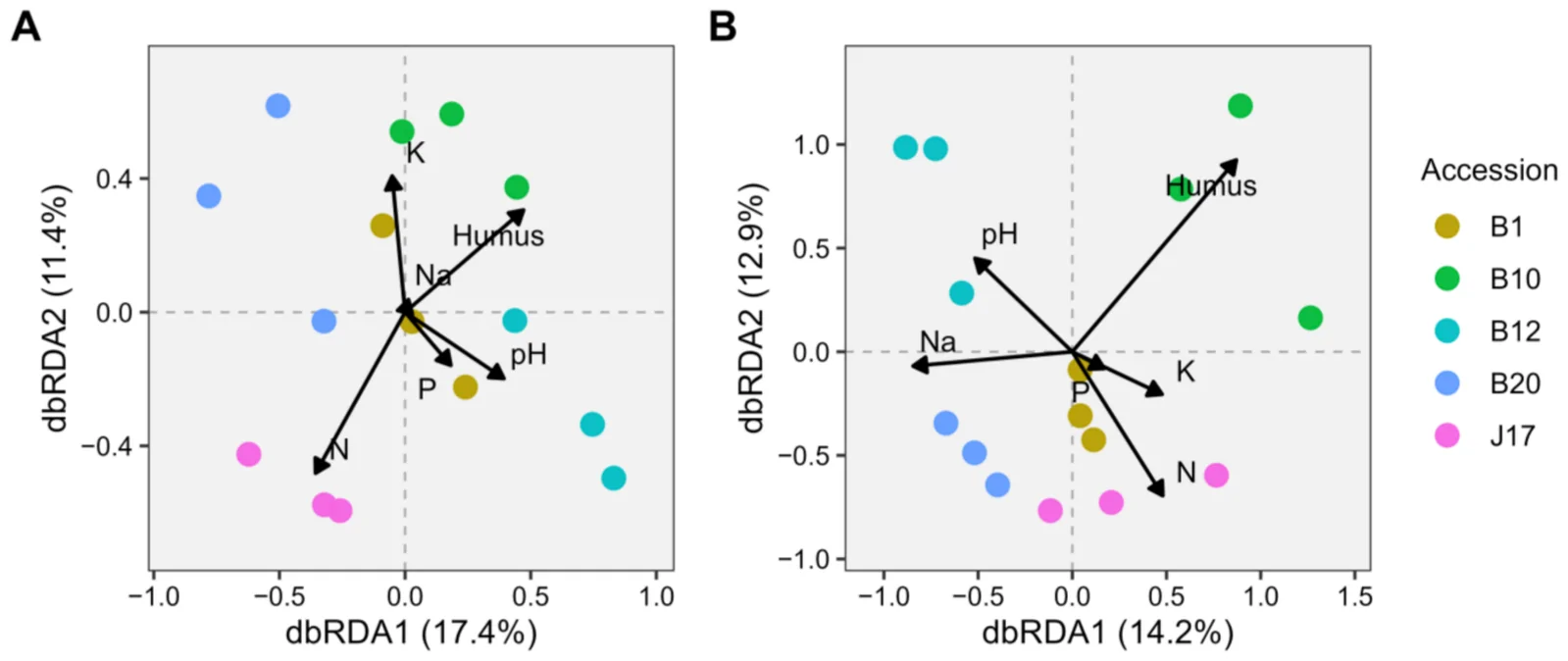
Associations between soil physicochemical properties and bacterial (**A**) and fungal (**B**) community composition in *Mentha* rhizosphere samples. dbRDA was based on Bray–Curtis dissimilarities and standardized soil variables. Arrows indicate the direction of associations with humus content, available P, K and Na, pH(KCl), and N (NO_3_ + NO_2_). B1, *M. spicata*; B10, *M.* × *villosa*; B12, *M. longifolia*; B20, *M.* × *piperita*; J17, *M. suaveolens*. Each point represents an individual rhizosphere soil sample from one of the five accession plots.

Distance-based redundancy analysis (dbRDA) was used to examine the relationship between soil physicochemical variation and microbial community composition within the 15 *Mentha* rhizosphere samples (Figure 5). The bacterial model included humus content, pH(KCl), Nitrate + nitrite nitrogen, and available phosphorus, potassium, and sodium. The overall model was significant and explained 52.86% of the variation in bacterial community composition (R^2^ = 0.5286, adjusted R^2^ = 0.175, F_6,8_ = 1.50, *p* = 0.021). The first two constrained axes accounted for 17.4% and 11.4% of the total bacterial community variation, respectively. Samples from the five sampled accession plots showed partial differentiation along these environmental gradients, although substantial overlap remained among groups. None of the individual soil variables showed a significant marginal effect after accounting for the remaining predictors in the model (all *p* > 0.05), with available potassium showing the strongest individual tendency (*p* = 0.083).

A similar pattern was observed for the fungal community. The overall dbRDA model was significant and explained 51.99% of the variation in fungal community composition (R^2^ = 0.520, adjusted R^2^ = 0.160, F_6,8_ = 1.44, *p* = 0.006). The first two constrained axes explained 14.2% and 12.9% of the total fungal community variation, respectively. As in the bacterial analysis, no individual soil parameter had a significant marginal effect when evaluated together with the other predictors. The lowest marginal *p*-values were observed for available potassium (*p* = 0.080) and humus content (*p* = 0.081).

## 3. Discussion

This study compared the rhizosphere bacterial and fungal communities associated with five *Mentha* accession plots, with particular emphasis on the extent to which their microbiomes were shared or variable across the different plant materials. The accessions were grown within the same experimental field under comparable management and without imposed experimental treatments, providing a relatively similar environmental background for the comparison. At the same time, the five *Mentha* accessions represented closely related but biochemically diverse plant materials, as illustrated by their markedly different aerial-tissue EO profiles.

### 3.1. Biochemical Context and EO Differentiation

The EOs of *M. spicata* B1 and *M.* × *villosa* B10 were both dominated by L-carvone, but they differed strongly in their other major components. Carvone-dominated profiles are common among cultivated *M. spicata* and have also been documented in *M.* × *villosa* [32,33]. In *M.* × *villosa* B10, limonene (the precursor of carvone) represented almost one quarter of the oil, whereas *M. spicata* B1 showed a more even contribution of trans-sabinene hydrate, 1,8-cineole and limonene. The presence of trans-sabinene hydrate (a thujyl derivative) as a major component in spearmint EO is not typical, but it occurs in some accessions. A spearmint EO from Turkey containing trans-sabinene hydrate (21.90%) and carvone (18.60%) has previously been reported [34]. The remaining accessions were characterized by distinctly different dominant compounds: cis- and trans-dihydrocarvone in *M. longifolia* B12, menthone and menthol in *M.* × *piperita* B20, and cis-piperitone epoxide in *M. suaveolens* J17. Dihydrocarvone isomers as dominant EO components are rare, but they were recently reported in a Northern Hungarian *M. longifolia* chemotype [35]. Menthol and menthone (a precursor of menthol) are typical in cultivated peppermint [36]. The presence of cis-piperitone epoxide in *M. suaveolens* EO is not unusual; however, the trans isomer appears more frequently as a dominant component, as summarized in a recent review [37]. Piperitone epoxides commonly co-occur with piperitenone oxide (syn. rotundifolone) in *M. suaveolens* EOs. The latter epoxide was also present here, but at a relatively low proportion. A chemotype containing a high proportion (44–55%) of cis-piperitone epoxide has also recently been reported in *M. longifolia*, a taxon closely related to *M. suaveolens* [35]. In that case, however, the epoxide was escorted by sesquiterpenes as major components.

The combination of close taxonomic relatedness, a common field background and marked biochemical differentiation provides a useful context for asking how much of the associated rhizosphere microbiome is conserved across these *Mentha* accession plots and how much belongs to a more variable community fraction.

### 3.2. Shared and Variable Fractions of Bacterial and Fungal Rhizosphere Communities

A clear difference emerged between bacterial and fungal communities in the extent of ASV sharing across the five sampled *Mentha* accession plots. Here, the shared fraction represents an operationally defined ASV pool reproducibly detected across the five sampled accession plots under the applied within-plot prevalence criterion. Under the primary ≥2/3 criterion, bacterial ASVs showed substantially greater sharing than fungal ASVs, and this contrast remained robust across alternative prevalence thresholds and after rarefaction. Taxonomic aggregation at genus and family levels reduced the magnitude of the difference, particularly for fungi, but did not eliminate it. Similar patterns were obtained whether unresolved taxonomic assignments were retained as operational bins or excluded from the fixed-rank analysis. Differences in taxonomic resolution may therefore contribute to the magnitude of the observed ASV-level contrast, particularly for fungi, but do not fully account for the substantially greater bacterial sharing. At the same time, overlap at genus or family level represents a coarser form of taxonomic similarity and does not imply identical species-, strain-, or sequence-level composition among samples. Direct quantitative comparison of 16S- and ITS-derived feature proportions should nevertheless be interpreted cautiously because the two marker systems differ in amplicon characteristics, primer-dependent amplification, taxonomic resolution, and detection properties, all of which can influence observed richness and prevalence.

Importantly, most of the ASVs shared across all five *Mentha* accession plots were also reproducibly detected in the managed inter-row soil. Of the 3005 shared bacterial ASVs, 2759 (91.81%) met the same ≥2/3 prevalence criterion in the managed inter-row soil, as did 169 of the 188 shared fungal ASVs (89.89%). The broadly shared fraction, particularly the very large bacterial component, should not be interpreted as a *Mentha*-specific conserved microbiome. Rather, a substantial part of it likely represents a common soil-associated microbial pool that was consistently detected both in the rhizosphere and in the managed inter-row reference. Consistent with this interpretation, only 91 bacterial ASVs met the separate operational definition of occurring in at least 12 of the 15 rhizosphere samples while being absent from all three managed inter-row soil samples, and these accounted for only 1.46% of bacterial reads. This rhizosphere-associated category reflects prevalence and absence in the reference samples and does not by itself demonstrate statistically significant rhizosphere enrichment or *Mentha*-specific selection.

Core estimates are inherently sensitive to prevalence thresholds and analytical choices, and no universally accepted criterion exists for separating core from variable community members [7,8]. Similar occupancy-abundance relationships have been observed in other plant systems, where consistently detected taxa can represent only part of the total taxonomic richness while accounting for a disproportionately large share of community abundance [9,10,38]. Across the five sampled accession plots, bacterial communities were consequently characterized by extensive ASV-level sharing, whereas the fungal communities contained an abundant shared component accompanied by a much larger pool of less consistently occurring ASVs.

Alpha-diversity patterns provided a complementary perspective. Bacterial alpha diversity differed primarily between the *Mentha* rhizosphere and managed inter-row soil, with no significant pairwise differences among the five accession plots, whereas fungal alpha-diversity patterns were more variable and only Shannon diversity differed significantly among groups. The extensive bacterial overlap did not imply identical communities among the five sampled accession plots. Beta-diversity analysis still detected significant structuring among the sampled *Mentha* accession plots, consistent with differentiation occurring largely through changes in the relative representation of taxa drawn from a broadly shared bacterial pool. A comparable pattern has been reported across six plant species grown in the same field, where a substantial shared bacterial rhizosphere core coexisted with significant plant-taxon-associated differences in community composition and diversity [39]. A similar tendency had already been suggested in our earlier three-*Mentha* comparison, where 94.7% of bacterial ASVs were shared across the three rhizosphere groups and managed inter-row soil, whereas fungal ASVs showed substantially less overlap [30]. The separate study of five *M. spicata* accession plots also showed a more compact bacterial community structure and greater variability among fungal samples [31]. The present analysis extends these earlier observations by examining a broader set of *Mentha* plant materials represented by five accession plots and by incorporating within-plot prevalence rather than relying solely on pooled group-level presence.

The greater fungal turnover was also apparent in the accession-plot-associated fraction, where exclusive ASVs were detected only for fungi and together accounted for less than 1% of fungal reads. Given their low cumulative abundance, these ASVs should be interpreted cautiously and primarily as components of the variable fungal fraction rather than as evidence of biologically important accession-specific signatures. Differences between bacterial and fungal community responses have been reported in a range of plant-associated systems, although the direction and magnitude of these differences are not universal [40]. Large-scale sampling of a medicinal-plant rhizosphere found stronger dispersal limitation and higher compositional heterogeneity for fungi than for bacteria [11], while cultivar-related effects in chrysanthemum were more strongly associated with bacterial than fungal community selection [12]. These observations argue against a simple interpretation based on greater host specificity of one microbial kingdom. In the present dataset, the extensive bacterial sharing is consistent with repeated recruitment from a broadly distributed soil-associated microbial pool, whereas the larger variable fungal fraction is compatible with stronger spatial turnover related to dispersal limitation, heterogeneous resource use, life-history differences, or greater sensitivity to local microsite conditions [5,11,12]. These mechanisms were not directly tested here and should therefore be regarded as plausible ecological interpretations rather than demonstrated causes.

### 3.3. Links Between Bacterial and Fungal Community Variation and Soil Properties

Despite these differences in the balance between shared and variable community fractions, variation in the bacterial and fungal communities was not independent. Bray–Curtis dissimilarities were moderately positively correlated, and this relationship remained significant when the analysis was restricted to the *Mentha* rhizosphere samples. This association does not demonstrate direct interactions between bacteria and fungi, but it indicates that some of the ecological variation among the rhizosphere samples was reflected in both kingdoms in parallel. Comparable cross-kingdom concordance has been reported in other rhizosphere systems, including medicinal plants [11] and willow cultivars, where bacterial and fungal Bray–Curtis dissimilarities were significantly correlated under non- and moderately contaminated field conditions [41]. Rhizosphere community structure generally reflects overlapping effects of soil conditions, plant characteristics, developmental state and microbial interactions rather than the action of a single environmental filter [5,6,42].

The overall dbRDA models based on soil physicochemical variables were significant for both bacterial and fungal communities, but none of the individual soil variables showed a significant marginal effect when evaluated together with the remaining predictors. This does not conflict with the large effect sizes observed for several soil parameters in the univariate ANOVAs, because the two analyses address different questions. The ANOVAs quantified differences in individual soil properties among all six sampled groups, including managed inter-row soil, whereas the dbRDA was restricted to the 15 Mentha rhizosphere samples and evaluated the unique association of each soil variable with multivariate microbial community composition while accounting for the remaining soil predictors. Covariation among soil properties, the narrower environmental range within the rhizosphere-only dataset, and the limited sample size relative to the number of predictors may therefore reduce the significance of individual marginal effects even when the overall multivariate model is significant. Given the comparatively uniform field setting and the absence of experimentally imposed soil treatments, a single dominant edaphic driver would not necessarily be expected. In contrast, broader spatial surveys of medicinal plants have identified environmental conditions, including soil pH, as major determinants of rhizosphere bacterial community variation, illustrating how the apparent importance of environmental drivers can depend strongly on sampling scale and environmental heterogeneity [43]. Similarly, across a geographic survey of wheat rhizospheres, pH, organic carbon and nutrient availability were associated with bacterial diversity and with the relative abundance of core taxa [44]. The measured soil variables instead describe local variation across the sampled accession plots within the same field environment, where nutrient availability, organic matter and pH may covary and act together with plant-associated and microsite-level processes. This pattern differs somewhat from the previous *M. spicata* accession study from the same experimental field, where pH and nitrate plus nitrite nitrogen showed significant marginal associations with both bacterial and fungal communities and humus content was additionally associated with fungal composition [31]. Stronger relationships between several soil properties and bacterial community characteristics were also detected in the earlier three-*Mentha* study [30]. Together, these related datasets suggest that soil chemistry contributes to microbial community variation, but the dominant predictors may differ among sampling contexts. Plant traits such as root architecture, resource uptake and root exudation can modify microbial recruitment within the environmental template provided by the surrounding soil [6,45].

### 3.4. Taxonomic Composition of Core and Variable ASV Fractions

The taxonomic composition of the shared communities was broadly consistent with previous studies of *Mentha*, other *Lamiaceae* and medicinal-plant rhizospheres. Actinomycetota, Pseudomonadota, Acidobacteriota and Chloroflexota were major components of the bacterial communities, while Ascomycota dominated the fungal assemblages together with Mortierellomycota and Basidiomycota [19,20,21,30,31,46,47]. More specifically, the bacterial core contained recurrent Gaiellales-, Vicinamibacterales- and *Nocardioidaceae*-affiliated ASVs, whereas the fungal core included members of *Nectriaceae*, *Didymellaceae*, *Chaetomiaceae*, *Plectosphaerellaceae*, *Mortierellaceae* and *Stachybotryaceae*. At genus level, repeatedly detected fungi included *Fusarium*, *Humicola*, *Plectosphaerella*, *Didymella* and *Tetracladium*. Their presence in the core should not be interpreted as evidence of a specific beneficial or pathogenic function, because genus-level assignments can encompass taxa with different ecological roles. This is particularly relevant for *Fusarium*, which includes both pathogenic and non-pathogenic plant-associated members [48].

### 3.5. Study Limitations

Several limitations should be considered when interpreting these findings. Each *Mentha* accession was represented by a single field plot, and the three samples collected within each plot captured spatial variation rather than independent plot-level replication. Differences among groups therefore describe patterns among the sampled accession plots and should not be interpreted as replicated accession- or species-level effects. Because the reference samples were collected from vegetation-free inter-row areas subjected to shallow mechanical cultivation, differences between the rhizosphere and managed inter-row soil may reflect both plant-associated and management- or disturbance-related effects and should not be interpreted as comparisons with undisturbed bulk soil. The study was conducted at a single field site and at one phenological stage, so the temporal stability and broader environmental generality of the identified core and variable fractions remain unknown. Amplicon sequencing primarily provides taxonomic information and does not directly establish functional roles, and estimates of core and shared-community membership are influenced by marker choice, sequencing depth, filtering, taxonomic resolution, and prevalence thresholds. Although the qualitative bacterial–fungal contrast remained after rarefaction, across alternative prevalence criteria, and after genus- and family-level aggregation, its magnitude was partly dependent on taxonomic resolution. In addition, only a limited set of soil physicochemical properties was measured; unmeasured plant-associated factors may also contribute to rhizosphere community structure.

## 4. Materials and Methods

### 4.1. Experimental Design and Sampling

Plant and soil samples were collected in late July 2025 at full flowering from the experimental field of the Department of Medicinal and Aromatic Plants, Hungarian University of Agriculture and Life Sciences. The experimental stands had been established in 2022 by vegetative propagation from mother plants. The study included five *Mentha* accessions: *M. spicata* B1, *M.* × *villosa* B10, *M. longifolia* B12, *M.* × *piperita* B20, and *M. suaveolens* J17. The accession codes correspond to entries in the institutional gene bank. Each accession was grown in a single 1.5 × 2.5 m plot, separated from adjacent plots by 1.5 m-wide inter-row strips. The inter-row areas were kept free of cultivated and spontaneous vegetation by mechanical weeding or shallow tillage. The field layout was not randomized or blocked, and independent plot-level replication was not available. In the present study, the term “accession” refers to the plant material identified by its gene-bank code, whereas “accession plot” refers to the single field plot occupied by that accession.

Three spatially separated sampling points were established within each accession plot, approximately 1 m apart in a roughly triangular arrangement. These samples are denoted by the suffixes a, b, and c throughout the study (e.g., B1a, B1b, and B1c). At each sampling point, an approximately 15 × 15 cm block containing vegetation, roots, and soil was excavated to a depth of 10–15 cm. Because the established mint stands were dense and interconnected by stolons, individual plants could not be separated reliably, and each sampling block represented a local section of the root system rather than a single plant. Soil released by shaking the excavated root system was collected without separating loosely and tightly root-adhering fractions. No separate rhizosphere and rhizoplane fractions were analyzed. This material is hereafter referred to as rhizosphere soil. The three samples from each accession plot were processed separately and represent within-plot spatial variation rather than independent plot-level biological replicates.

Three additional soil samples were collected from vegetation-free inter-row areas approximately 50 cm from the nearest *Mentha* rows. Because these areas were regularly subjected to shallow mechanical cultivation, they represented managed inter-row soil rather than undisturbed natural soil. These samples are therefore referred to throughout the manuscript as managed inter-row soil; the sample identifiers Bulk_a–Bulk_c are retained for consistency with the sequencing dataset. Sampling tools were disinfected with 70% ethanol between sampling points, and a new pair of disposable gloves was used at each location. Soil samples were transferred to sterile 50 mL tubes, transported to the laboratory on ice, and processed upon arrival. After thorough mixing and removal of visible roots and plant debris, aliquots for DNA extraction were placed in sterile 1.5 mL tubes and stored at −80 °C.

The sequence datasets from *M. spicata* B1 and managed inter-row soil had previously been included in a separate analysis of *M. spicata* accession plots [31]. In the present study, these data were analyzed jointly with the *M.* × *villosa* B10, *M. longifolia* B12, *M.* × *piperita* B20, and *M. suaveolens* J17 datasets in the current comparison across the five accession plots.

### 4.2. Soil Physicochemical Analyses

The three rhizosphere soil samples from each accession plot and the three managed inter-row soil samples were analyzed separately, with one determination per parameter for each soil sample. Soil physicochemical analyses followed standard Hungarian soil analytical methods. Soil texture classification was based on the Arany-type plasticity index (MSZ 08-0205:1978). Soil pH was determined in 1 M KCl suspension according to MSZ 08-0206:1978, while humus content was measured by potassium dichromate oxidation following MSZ 21470-52:1983. Available phosphorus (P_2_O_5_), potassium (K_2_O), and sodium (Na^+^) were extracted using ammonium lactate solution and determined according to MSZ 20135:1999. Nitrate + nitrite nitrogen (NO_3_^−^ + NO_2_^−^) was extracted with 1 M KCl and quantified according to the same standard. The reported P_2_O_5_, K_2_O, and Na^+^ concentrations represent agronomically available or exchangeable nutrient fractions.

### 4.3. Essential Oil Extraction and GC-MS Analysis

Approximately 200 g of flowering shoots, about 40 cm in length, were harvested from each accession and shade-dried for three weeks. After drying, leaves were separated from the stems and used for hydrodistillation. For each accession, three separate 10 g portions of dried leaf material were distilled with 500 mL distilled water for 2.5 h using a Clevenger-type apparatus. Residual water was removed from the resulting EOs with anhydrous sodium sulfate, and the oils were stored in airtight vials at 4 °C until GC-MS analysis.

EOs were analyzed using an Agilent Technologies 6890N gas chromatograph (Agilent Technologies, Inc., Santa Clara, CA, USA) coupled to a 5975 inert mass selective detector and fitted with an HP-5MS capillary column (30 m × 0.25 mm i.d., 0.25 µm film thickness). The oven temperature was initially set to 60 °C and increased at 3 °C/min to 240 °C, followed by a 5 min hold at the final temperature. Helium was used as carrier gas at a constant flow rate of 1 mL/min. Injector and detector temperatures were 250 °C, and the split ratio was 30:1. EOs were diluted in *n*-hexane at 10 µL oil per 1 mL solvent, and 0.2 µL of the diluted sample was injected. Linear retention indices (LRI) were calculated relative to a homologous series of C9–C23 *n*-alkanes using the equation of Van den Dool and Kratz (1963) [49]. Compound identification was based on retention-index comparison with literature data and mass-spectral matching against the NIST and Wiley libraries.

### 4.4. DNA Extraction and Amplicon Sequencing

Genomic DNA was extracted separately from 100 mg of each rhizosphere and managed inter-row soil sample using the ZymoBIOMICS 96 MagBead DNA Kit (Zymo Research, Irvine, CA, USA) according to the manufacturer’s instructions. DNA concentration was measured with the Quant-iT dsDNA BR Assay Kit (Thermo Fisher Scientific, Waltham, MA, USA) using an Infinite Pro 200 F Nano+ fluorescent plate reader (Tecan, Männedorf, Switzerland).

Bacterial and fungal communities were characterized by amplicon sequencing of the bacterial 16S rRNA gene and fungal internal transcribed spacer (ITS) region, respectively. Library preparation and sequencing were performed by Xenovea Kft. (Szeged, Hungary). The V3–V4 region of the bacterial 16S rRNA gene was amplified using tagged primers described by Klindworth et al. (2013) [50], whereas fungal ITS amplicons were generated using tagged ITS1F and ITS4R primers [51]. For the first amplification step, 50 ng template DNA was amplified with KAPA HiFi HotStart Ready Mix (Roche AG, Basel, Switzerland) for 25 cycles. After purification, 10 ng of the PCR product was used for indexing with the Nextera XT Index v2 Kit (Illumina, San Diego, CA, USA) and KAPA HiFi HotStart Ready Mix for eight cycles. Indexed libraries were purified using 0.8× volume KAPA Pure Beads (Roche AG, Basel, Switzerland). Libraries were quantified with the Equalbit 1× dsDNA HS Assay Kit (Vazyme Biotech, Nanjing, China), and fragment-size distributions were checked by capillary electrophoresis using a LabChip GX Touch HT Nucleic Acid Analyzer with the DNA NGS 3K Assay Kit (Revvity, Waltham, MA, USA). Libraries were normalized to equimolar concentrations, pooled, and sequenced on an Illumina NextSeq 2000 platform using 2 × 300 bp paired-end chemistry.

### 4.5. Bioinformatic Processing

Raw reads were demultiplexed and adapter-trimmed using the NextSeq Control Software (Illumina, Inc., San Diego, CA, USA). Reads with a mean quality score below 30 were removed using the FastQ Toolkit (Illumina, Inc., San Diego, CA, USA). Paired-end 16S and ITS reads were processed using the FROGS pipeline v4.2.0 [52]. Forward and reverse reads were quality-filtered and merged with VSEARCH v2.17.0 [53], retaining merged amplicons between 150 and 600 bp and allowing a mismatch rate of 0.1. Merged sequences were clustered with SWARM v3.0.0 [54], using an aggregation distance of one nucleotide (*d* = 1) followed by fastidious refinement. Chimeric sequences were identified and removed separately within each sample using the remove_chimera.py procedure implemented in FROGS.

Throughout this study, the term ASV is used according to the terminology of the FROGS output and refers to the sequence clusters generated by the SWARM-based workflow. These ASVs should therefore not be interpreted as exact sequence variants inferred using a denoising algorithm. Clusters representing less than 0.005% of the total sequence count were removed, and no further abundance- or prevalence-based filtering was applied during the primary bioinformatic processing.

Fungal sequences were screened using ITSx v1.1.2 with the Fungi profile while retaining conserved regions. For the bacterial dataset, only clusters assigned to Bacteria or Archaea were retained, while chloroplast, mitochondrial, and other non-target sequences were excluded. Taxa that could not be resolved at lower taxonomic levels were retained and labeled according to their lowest reliably assigned rank. Taxonomic classification was carried out with BLAST v2.16.0 [55] against SILVA 138.2 [56] for bacterial 16S rRNA sequences and UNITE Fungi 10.0 [57] for fungal ITS sequences. No additional species-level confidence threshold was applied.

The filtered, non-rarefied ASV abundance tables were retained for taxonomic composition and prevalence-based analyses. Before alpha- and beta-diversity calculations, the abundance tables were rarefied to the lowest sequencing depth within each dataset, corresponding to 112,125 sequences per sample for the bacterial dataset and 75,556 sequences per sample for the fungal dataset. No samples were excluded during rarefaction.

### 4.6. Statistical Analysis

Statistical analyses were performed in R version 4.4.3 [58], with *p* < 0.05 considered statistically significant. Because each *Mentha* accession was represented by a single field plot and the three samples within each plot represented spatial subsamples, statistical comparisons among accession groups were interpreted as differences among the sampled accession plots rather than as replicated accession- or species-level treatment effects.

Soil physicochemical parameters were compared among the five *Mentha* accession plots and managed inter-row soil using one-way ANOVA, followed by Tukey’s HSD post hoc test. Effect sizes were expressed as eta squared (η^2^), calculated as the proportion of total variance attributable to the group effect. Data are presented as mean ± standard deviation (SD). EO composition was evaluated descriptively and is presented as mean ± SD; no inferential comparisons among accessions were performed.

Alpha-diversity indices (Observed ASVs, Chao1, Shannon, and inverse Simpson) were calculated from the rarefied ASV abundance tables. Differences among the five *Mentha* accession plots and managed inter-row soil were evaluated by one-way ANOVA followed by Tukey’s HSD post hoc test. Beta diversity was assessed using Bray–Curtis dissimilarities and visualized by principal coordinate analysis. Differences in community composition were tested by PERMANOVA using the adonis2 function in the vegan package (v2.7.3) with 9999 permutations. PERMANOVA was performed both for all six groups and separately for the five *Mentha* accession plots after exclusion of managed inter-row soil. Homogeneity of multivariate dispersion was assessed using betadisper followed by permutation testing with 9999 permutations. Concordance between bacterial and fungal community variation was further evaluated by Mantel tests comparing the corresponding Bray–Curtis dissimilarity matrices using Spearman correlation and 9999 permutations. Mantel tests were performed for all 18 samples and separately for the 15 *Mentha* rhizosphere samples.

Core and shared ASV analyses were performed using the filtered, non-rarefied abundance tables, with an ASV considered present when its read count was >0. The primary rhizosphere core comprised ASVs detected in at least 12 of the 15 *Mentha* rhizosphere samples, while the strict rhizosphere core required detection in all 15 samples and the universal core required detection in all 18 (15 rhizosphere and three managed inter-row soil) samples. For accession-plot-level analyses, an ASV was considered present within an accession plot when detected in at least two of its three spatial samples; ASVs meeting this criterion in all five accession plots constituted the shared fraction across the five accession plots. For this shared fraction, we additionally recorded the number of ASVs that also met the same ≥2/3 prevalence criterion in the managed inter-row soil group. Sensitivity analyses were performed using ≥1/3 and 3/3 within-plot prevalence thresholds. Rhizosphere-associated ASVs were defined as those detected in at least 12 of the 15 rhizosphere samples but absent from all three managed inter-row soil samples. This operational category was used to distinguish ASVs not detected in the managed inter-row reference and should not be interpreted as evidence of rhizosphere enrichment or *Mentha*-specific selection, neither of which was directly tested. Accession-plot-associated exclusive ASVs were detected in at least two of three samples from one accession plot and absent from all samples from the other *Mentha* accession plots and from all managed inter-row soil samples. For each category, ASV numbers and cumulative relative abundances across the 15 rhizosphere samples were calculated. As an additional sensitivity analysis, the core and shared-community calculations were repeated using the rarefied abundance tables, applying the same prevalence criteria as in the primary analysis. To evaluate whether the bacterial–fungal contrast in shared-community size depended on ASV-level resolution, additional fixed-rank sensitivity analyses were performed at genus and family levels using the filtered, non-rarefied abundance tables. ASV counts were aggregated according to their genus or family assignments, and the same ≥2/3 within-plot prevalence criterion used in the primary ASV-level analysis was applied to the resulting taxonomic units. Two complementary treatments of unresolved assignments were evaluated. In the resolved-only analysis, ASVs lacking an assignment at the target taxonomic rank were excluded before aggregation. In the all-label analysis, all ASVs were retained; resolved assignments were aggregated by taxon, whereas ASVs sharing the same “unclassified_” label were combined into a single operational taxonomic bin representing the lowest reliably assigned higher-level lineage. These unresolved bins were treated as operational units rather than formally identified genera or families. Shared taxonomic units were defined as those meeting the ≥2/3 prevalence criterion in all five accession plots. Cumulative relative abundances were calculated across the 15 rhizosphere samples; for the resolved-only analysis, abundances were calculated relative to the retained rank-resolved read pool, whereas the all-label analysis retained the complete read pool.

Associations between soil physicochemical properties and microbial community composition were evaluated using dbRDA based on Bray–Curtis dissimilarities. These analyses were restricted to the 15 *Mentha* rhizosphere samples to assess environmental associations within the rhizosphere independently of the broader rhizosphere-managed inter-row soil contrast. Humus content, pH(KCl), nitrate plus nitrite nitrogen, available phosphorus (reported as P_2_O_5_), available potassium (reported as K_2_O), and available sodium were standardized before analysis. Multicollinearity was assessed using variance inflation factors, with a threshold of 10; all predictors were below this threshold and were retained. Models were fitted using capscale in the vegan package with Lingoes correction. Overall model significance, constrained axes, and marginal effects of individual soil variables were evaluated using 9999 permutations, and R^2^ and adjusted R^2^ values were calculated.

### 4.7. Use of Generative Artificial Intelligence

During the preparation of this manuscript, the authors used ChatGPT (OpenAI, San Francisco, CA, USA; GPT-5.6 Sol) to assist with language editing, improving clarity and readability, and refining the wording of selected manuscript sections based on author-provided content. The authors reviewed and edited all Artificial Intelligence-assisted output and take full responsibility for the content of this publication.

## 5. Conclusions

The rhizosphere microbiomes sampled from the five *Mentha* accession plots showed a clear contrast in the organization of bacterial and fungal communities. Bacterial communities were dominated by a broadly shared ASV pool, whereas fungal communities contained a smaller shared fraction together with substantially greater ASV-level variability. Because most ASVs shared across the five accession plots were also reproducibly detected in managed inter-row soil, this shared fraction should not be interpreted as a *Mentha*-specific conserved microbiome. This bacterial–fungal contrast remained consistent across alternative prevalence thresholds and after rarefaction, and following taxonomic aggregation at genus and family levels, supporting the robustness of the observed pattern. At the same time, bacterial and fungal community variation was significantly concordant, and both communities showed significant overall associations with measured soil physicochemical variation, although no individual soil variable had a significant marginal effect. Because each plant material was represented by only one field plot, these patterns cannot be separated from plot-level variation or generalized as replicated accession- or species-level effects. Overall, the results show that, within the sampled field setting, bacterial and fungal rhizosphere communities can differ markedly in the balance between broadly shared and more variable community components.

## Figures and Tables

**Table 1:** Physicochemical properties of rhizosphere soil from five *Mentha* accession plots and managed inter-row soil.

Parameter	B1	B10	B12	B20	J17	Bulk
Humus content (%)	2.18 ± 0.16 b	2.46 ± 0.09 a	2.18 ± 0.06 b	2.00 ± 0.09 bc	2.08 ± 0.03 bc	1.86 ± 0.07 c
pH (KCl)	7.96 ± 0.03 b	7.93 ± 0.02 b	7.96 ± 0.03 b	7.93 ± 0.01 b	7.93 ± 0.01 b	8.02 ± 0.02 a
(NO_3_^−^ + NO_2_^−^) (mg/kg)	2.52 ± 0.09 b	3.57 ± 0.47 b	1.64 ± 0.09 b	1.80 ± 0.36 b	17.27 ± 2.25 a	1.91 ± 0.05 b
Available Na^+^ (mg/kg)	51.27 ± 4.95 a	43.57 ± 4.19 a	54.27 ± 7.86 a	56.00 ± 8.79 a	41.70 ± 2.43 a	52.03 ± 3.76 a
Available P (P_2_O_5_ mg/kg)	740.67 ± 29.57 a	625.33 ± 21.59 ab	610.33 ± 89.19 b	582.33 ± 24.68 b	625.00 ± 42.93 ab	693.00 ± 26.89 ab
Available K (K_2_O mg/kg)	226.00 ± 25.51 a	162.00 ± 24.98 b	83.80 ± 11.61 d	136.33 ± 9.87 bc	88.77 ± 8.55 d	111.33 ± 6.66 cd

Values represent means ± standard deviation based on three soil samples for each accession plot and for managed inter-row soil (*n* = 3). Different lowercase letters within a row indicate significant differences among groups according to one-way analysis of variance (ANOVA) followed by Tukey’s honestly significant difference (HSD) test (*p* < 0.05). B1, *M. spicata*; B10, *M.* × *villosa*; B12, *M. longifolia*; B20, *M.* × *piperita*; J17, *M. suaveolens*.

**Table 2:** Core, shared, rhizosphere-associated, and accession-plot-associated bacterial and fungal ASV fractions in *Mentha* rhizosphere samples.

Category	16S ASVs *n* (%)	16S Cumulative Abundance (%)	ITS ASVs *n* (%)	ITS Cumulative Abundance (%)
Total filtered ASVs	3128 (100)	–	859 (100)	–
Universal core, 18/18	2158 (68.99)	86.04 ± 1.88	68 (7.92)	51.29 ± 8.65
Strict rhizosphere core, 15/15	2762 (88.30)	96.00 ± 1.04	93 (10.83)	58.80 ± 9.48
Rhizosphere core, ≥12/15	3062 (97.89)	99.33 ± 0.40	243 (28.29)	78.47 ± 5.72
Shared fraction across all five *Mentha* accession plots, ≥2/3 each	3005 (96.07)	98.65 ± 0.52	188 (21.89)	72.83 ± 7.66
Rhizosphere-associated, ≥12/15 and absent from managed inter-row soil	91 (2.91)	1.46 ± 0.54	13 (1.51)	2.59 ± 5.45
Accession-plot-associated exclusive	0	0	29 (3.38)	0.79 ± 0.67

ASV counts are reported as absolute numbers (*n*), with the corresponding percentage of the total ASV pool given in parentheses. Cumulative abundance represents, for each rhizosphere sample, the summed relative abundance of all ASVs assigned to the respective category; values are reported as the mean ± SD across all samples. The ≥80% rhizosphere core includes ASVs detected in at least 12 of 15 rhizosphere samples, while the strict rhizosphere core includes ASVs detected in all 15 samples. The shared fraction across the five accession plots comprises ASVs detected in at least two of the three spatial samples within each of the five *Mentha* accession plots. Rhizosphere-associated ASVs were detected in at least 12 of 15 rhizosphere samples but were absent from all three managed inter-row soil samples. Accession-plot-associated exclusive ASVs were detected in at least two of three samples of a single accession plot and were absent from all other *Mentha* and managed inter-row soil samples.

## Data Availability

The raw bacterial 16S rRNA gene and fungal ITS amplicon sequencing data analyzed in this study are available in the NCBI Sequence Read Archive. The *M. spicata* B1 and bulk soil datasets have been deposited under BioProject accession number PRJNA1465696, while the sequencing data for *M.* × *villosa* B10, *M. longifolia* B12, *M.* × *piperita* B20, and *M. suaveolens* J17 have been deposited under a separate BioProject accession number PRJNA1524358. All other data supporting the findings of this study are included in the article and its Supplementary Materials.

## Supplementary Materials

The following supporting information can be downloaded at https://www.mdpi.com/article/10.3390/plants15203081/s1, Table S1: Essential oil composition of the analyzed *Mentha* accessions; Table S2: Summary of bacterial 16S rRNA gene sequencing data and taxonomic richness across *Mentha* rhizosphere and managed inter-row soil samples; Table S3: Summary of fungal ITS gene sequencing data and taxonomic richness across *Mentha* rhizosphere and managed inter-row soil samples; Table S4: Alpha-diversity indices of bacterial communities across the five *Mentha* accession plots and managed inter-row soil; Table S5: Alpha-diversity indices of fungal communities across the five *Mentha* accession plots and managed inter-row soil; Table S6: Sensitivity analysis of core and shared bacterial and fungal ASV fractions before and after rarefaction; Table S7: Fixed-rank sensitivity analysis of shared bacterial and fungal community fractions and the effect of unresolved taxonomic assignments; Table S8: Representative taxa within core, shared, rhizosphere-associated, and accession-plot-associated exclusive ASV fractions; Figure S1: UpSet analysis of shared bacterial and fungal ASVs using the ≥1/3 within-group prevalence criterion. Exact intersections of bacterial 16S rRNA gene ASVs (A) and fungal ITS ASVs (B) are shown among the five *Mentha* accession plots and managed inter-row soil. An ASV was considered present in an accession plot or the managed inter-row soil group when detected in at least one of the three spatial samples. Horizontal bars indicate set sizes, and vertical bars show the 20 largest exact intersections; Figure S2: UpSet analysis of shared bacterial and fungal ASVs using the 3/3 within-group prevalence criterion. Exact intersections of bacterial 16S rRNA gene ASVs (A) and fungal ITS ASVs (B) are shown among the five *Mentha* accession plots and managed inter-row soil. An ASV was considered present in an accession plot or the managed inter-row soil group when detected in all three spatial samples. Horizontal bars indicate set sizes, and vertical bars show the 20 largest exact intersections.

## Abbreviations

The following abbreviations are used in this manuscript:

16S rRNA	16S ribosomal RNA
ANOVA	Analysis of variance
ASV	Amplicon sequence variant
BLAST	Basic Local Alignment Search Tool
dbRDA	Distance-based redundancy analysis
EO	Essential oil
FROGS	Find, Rapidly, OTUs with Galaxy Solution
GC-MS	Gas chromatography–mass spectrometry
HSD	Honestly significant difference
ITS	Internal transcribed spacer
LRI	Linear retention index
MSZ	Hungarian Standard (Magyar Szabvány)
NCBI	National Center for Biotechnology Information
NGS	Next-generation sequencing
NIST	National Institute of Standards and Technology
PCoA	Principal coordinate analysis
PCR	Polymerase chain reaction
PERMANOVA	Permutational multivariate analysis of variance
PERMDISP	Permutational analysis of multivariate dispersions
SD	Standard deviation

## References

[1] Berendsen R.L., Pieterse C.M., Bakker P.A. (2012). The rhizosphere microbiome and plant health. Trends Plant Sci..

[2] Mendes R., Garbeva P., Raaijmakers J.M. (2013). The rhizosphere microbiome: Significance of plant beneficial, plant pathogenic, and human pathogenic microorganisms. FEMS Microbiol. Rev..

[3] Chaparro J.M., Badri D.V., Vivanco J.M. (2014). Rhizosphere microbiome assemblage is affected by plant development. ISME J..

[4] Sasse J., Martinoia E., Northen T. (2018). Feed your friends: Do plant exudates shape the root microbiome?. Trends Plant Sci..

[5] Trivedi P., Leach J.E., Tringe S.G., Sa T., Singh B.K. (2020). Plant–microbiome interactions: From community assembly to plant health. Nat. Rev. Microbiol..

[6] Negre Rodríguez M., Pioppi A., Kovács Á.T. (2025). The role of plant host genetics in shaping the composition and functionality of rhizosphere microbiomes. Msystems.

[7] Risely A. (2020). Applying the core microbiome to understand host–microbe systems. J. Anim. Ecol..

[8] Neu A.T., Allen E.E., Roy K. (2021). Defining and quantifying the core microbiome: Challenges and prospects. Proc. Natl. Acad. Sci. USA.

[9] Simonin M., Dasilva C., Terzi V., Ngonkeu E.L., Diouf D., Kane A., Béna G., Moulin L. (2020). Influence of plant genotype and soil on the wheat rhizosphere microbiome: Evidences for a core microbiome across eight African and European soils. FEMS Microbiol. Ecol..

[10] Wang B., Chen C., Xiao Y., Chen K., Wang J., Wang L., Li J., Kang Z., Zhou G. (2023). A core root bacteria contribute to plant growth and anisodine accumulation of Anisodus tanguticus. BMC Plant Biol..

[11] Zhang G., Wei G., Wei F., Chen Z., He M., Jiao S., Wang Y., Dong L., Chen S. (2021). Dispersal limitation plays stronger role in the community assembly of fungi relative to bacteria in rhizosphere across the arable area of medicinal plant. Front. Microbiol..

[12] Rotoni C., Leite M.F.A., Pijl A., Kuramae E.E. (2022). Rhizosphere microbiome response to host genetic variability: A trade-off between bacterial and fungal community assembly. FEMS Microbiol. Ecol..

[13] Salehi B., Stojanović-Radić Z., Matejić J., Sharopov F., Antolak H., Kręgiel D., Sen S., Sharifi-Rad M., Acharya K., Sharifi-Rad R. (2018). Plants of genus Mentha: From farm to food factory. Plants.

[14] Zhang J., Li M., Zhang H., Pang X. (2022). Comparative investigation on aroma profiles of five different mint (*Mentha*) species using a combined sensory, spectroscopic and chemometric study. Food Chem..

[15] Moetamedipoor S.A., Saharkhiz M.J., Khosravi A.R., Jowkar A. (2021). Essential oil chemical diversity of Iranian mints. Ind. Crops Prod..

[16] Adamović D., Đalović I., Mrkovački N. (2015). Microbial abundance in rhizosphere of medicinal and aromatic plant species in conventional and organic growing systems. Ratar. I Povrtarstvo/Field Veg. Crops Res..

[17] Santoro M.V., Bogino P.C., Nocelli N., Cappellari L., Giordano W.F., Banchio E. (2016). Analysis of plant growth-promoting effects of fluorescent Pseudomonas strains isolated from *Mentha piperita* rhizosphere and effects of their volatile organic compounds on essential oil composition. Front. Microbiol..

[18] Misra P., Maji D., Awasthi A., Pandey S.S., Yadav A., Pandey A., Saikia D., Babu C.S.V., Kalra A. (2019). Vulnerability of soil microbiome to monocropping of medicinal and aromatic plants and its restoration through intercropping and organic amendments. Front. Microbiol..

[19] Zharkova E.K., Vankova A.A., Selitskaya O.V., Malankina E.L., Drenova N.V., Zhelezova A.D., Khlyustov V.K., Belopukhov S.L., Zhevnerov A.V., Sviridova L.A. (2023). Bacterial communities of *Lamiacea* L. Medicinal Plants: Structural features and rhizosphere effect. Microorganisms.

[20] Applebaum I., Jeyaraman M., Sherman C., Doniger T., Steinberger Y. (2022). Structure and function of the soil rhizosphere fungal communities in medicinal plants—A preliminary study. Agriculture.

[21] Steinberger Y., Doniger T., Sherman C., Jeyaraman M., Applebaum I. (2024). Soil bacterial community of medicinal plant rhizosphere in a mediterranean system. Agriculture.

[22] Raveau R., Fontaine J., Hijri M., Lounes-Hadj Sahraoui A. (2020). The aromatic plant clary sage shaped bacterial communities in the roots and in the trace element-contaminated soil more than mycorrhizal inoculation—A two-year monitoring field trial. Front. Microbiol..

[23] Raveau R., Lounes-Hadj Sahraoui A., Hijri M., Fontaine J. (2021). Clary sage cultivation and mycorrhizal inoculation influence the rhizosphere fungal community of an aged trace-element polluted soil. Microorganisms.

[24] Jamwal V.L., Rather I.A., Ahmed S., Kumar A., Gandhi S.G. (2023). Changing rhizosphere microbial community and metabolites with developmental stages of *Coleus barbatus*. Microorganisms.

[25] Li L., Huang J., Liu Y., Zhang Q., Han Q., Liu Y., Zhang G., Wang X., Zhao W., Liu L. (2025). Analysis of microbial community composition and diversity in the rhizosphere of *Salvia miltiorrhiza* at different growth stages. Int. Microbiol..

[26] Deng X., Shi R., Elnour R.O., Guo Z., Wang J., Liu W., Li G., Jiao Z. (2024). Analysis of rhizosphere fungal diversity in lavender at different planting years based on high-throughput sequencing technology. PLoS ONE.

[27] Meneguzzi R., Fernandez M., Cappellari L., Giordano W., Banchio E. (2024). Isolation and characterization of plant growth-promoting bacteria from the rhizosphere of medicinal and aromatic plant *Minthostachys verticillata*. Plants.

[28] Checcucci A., Maida I., Bacci G., Ninno C., Bilia A.R., Biffi S., Firenzuoli F., Flamini G., Fani R., Mengoni A. (2017). Is the plant-associated microbiota of *Thymus* spp. adapted to plant essential oil?. Res. Microbiol..

[29] Li Y., Wang Y., Yan F., Guo Q., Tang J., Hu X., Chen Y., Yan Y., Gao J., Zhang G. (2025). 16S rDNA sequencing and targeted metabolomics reveal rhizosphere microbial diversity and flavonoid dynamics in *Scutellaria baicalensis* Georgi. BMC Microbiol..

[30] Wazzani Y., Tavaszi-Sárosi S., Patonay K., Olasz F., Juhász Á., Posta K. (2026). Microbial communities in the rhizosphere of three *Mentha* species: Links to soil properties and essential oil profiles. PLoS ONE.

[31] Juhász Á., Wazzani Y., Tavaszi-Sárosi S., Patonay K., Olasz F., Posta K. (2026). Rhizosphere bacterial and fungal community structure varies among field-grown *Mentha* spicata accession plots with different essential oil profiles. Front. Microbiol..

[32] Kowalczyk A., Piątkowska E., Kuś P., Marijanović Z., Jerković I., Tuberoso C.I., Fecka I. (2021). Volatile compounds and antibacterial effect of commercial mint cultivars-chemotypes and safety. Ind. Crops Prod..

[33] De Pooter H., Schamp N. (1987). The essential oil of *Mentha* × *villosa* nm. Alopecuroides Flavour Fragr. J..

[34] Baser K., Kürkçüoglu M., Tarimcilar G., Kaynak G. (1999). Essential oils of *Mentha* species from Northern Turkey. J. Essent. Oil Res..

[35] Patonay K., Szalontai H., Radácsi P., Zámboriné-Németh É. (2021). Chemotypes and their stability in *Mentha longifolia* (L.) L.—A comprehensive study of five accessions. Plants.

[36] Lawrence B.M. (2007). The composition of commercially important mints. Mint The Genus Mentha.

[37] Tavaszi-Sárosi S., Sfaxi A., Juhász Á., Radácsi P., Patonay K. (2025). Chemical and biological properties of *Mentha* × *villosa* Huds. (mojito mint) and its parental species—*Mentha spicata* L. (spearmint) and *Mentha suaveloens* Ehrh. (apple mint)—A review. Phytochem. Rev..

[38] Stopnisek N., Shade A. (2021). Persistent microbiome members in the common bean rhizosphere: An integrated analysis of space, time, and plant genotype. ISME J..

[39] Lei S., Xu X., Cheng Z., Xiong J., Ma R., Zhang L., Yang X., Zhu Y., Zhang B., Tian B. (2019). Analysis of the community composition and bacterial diversity of the rhizosphere microbiome across different plant taxa. Microbiologyopen.

[40] Li X., Zhang Y., Zhou C., Li X., Zou X., Ou L., Tao Y. (2024). The changes of rhizosphere microbial communities in pepper varieties with different capsaicinoids. Front. Microbiol..

[41] Bell T.H., El-Din Hassan S., Lauron-Moreau A., Al-Otaibi F., Hijri M., Yergeau E., St-Arnaud M. (2014). Linkage between bacterial and fungal rhizosphere communities in hydrocarbon-contaminated soils is related to plant phylogeny. ISME J..

[42] Wu J., Bai R., Akhberdi O., Xu L. (2026). Comparative analysis of rhizobacterial communities across five medicinal plants in Xinjiang. Front. Microbiol..

[43] Dong C., Shao Q., Ren Y., Ge W., Yao T., Hu H., Huang J., Liang Z., Han Y. (2022). Assembly, core microbiota, and function of the rhizosphere soil and bark microbiota in Eucommia ulmoides. Front. Microbiol..

[44] Kumar M., Ansari W.A., Zeyad M.T., Singh A., Chakdar H., Kumar A., Farooqi M.S., Sharma A., Srivastava S., Srivastava A.K. (2023). Core microbiota of wheat rhizosphere under Upper Indo-Gangetic plains and their response to soil physicochemical properties. Front. Plant Sci..

[45] Qu P., Wang B., Qi M., Lin R., Chen H., Xie C., Zhang Z., Qiu J., Du H., Ge Y. (2024). Medicinal plant root exudate metabolites shape the rhizosphere microbiota. Int. J. Mol. Sci..

[46] Vélez-Martínez G.A., Duque-Zapata J.D., Reyes-Ardila W.L., Muñoz Flórez J.E., Díaz Gallo S.A., Díaz Ariza L.A., López-Álvarez D. (2023). Comparative analysis of bacteria, fungi, and arbuscular mycorrhizal fungi in medicinal plants *Lippia alba* and *Petiveria alliacea* in Colombia. Diversity.

[47] Lu Y., Cao X., Qiao C., Li Y., Bai J., Zhang T., Cao Y., Zhao C., Cao Y., Zhao C. (2026). Rhizosphere microbiome assemblage impacts soil chemical properties and medicinal quality in *Atractylodes chinensis*. Front. Microbiol..

[48] Zhang J., Wei Y., Li H., Hu J., Zhao Z., Wu Y., Yang H., Li J., Zhou Y. (2023). Rhizosphere microbiome and phenolic acid exudation of the healthy and diseased American ginseng were modulated by the cropping history. Plants.

[49] Van Den Dool H., Kratz P.D. (1963). A generalization of the retention index system including linear temperature programmed gas-liquid partition chromatography. J. Chromatogr..

[50] Klindworth A., Pruesse E., Schweer T., Peplies J., Quast C., Horn M., Glöckner F.O. (2013). Evaluation of general 16S ribosomal RNA gene PCR primers for classical and next-generation sequencing-based diversity studies. Nucleic Acids Res..

[51] Illumina, Inc. (2019). Fungal Metagenomic Sequencing Demonstrated Protocol.

[52] Escudié F., Auer L., Bernard M., Mariadassou M., Cauquil L., Vidal K., Maman S., Hernandez-Raquet G., Combes S., Pascal G. (2018). FROGS: Find, rapidly, OTUs with galaxy solution. Bioinformatics.

[53] Rognes T., Flouri T., Nichols B., Quince C., Mahé F. (2016). VSEARCH: A versatile open source tool for metagenomics. PeerJ.

[54] Mahé F., Rognes T., Quince C., de Vargas C., Dunthorn M. (2014). Swarm: Robust and fast clustering method for amplicon-based studies. PeerJ.

[55] McGinnis S., Madden T.L. (2004). BLAST: At the core of a powerful and diverse set of sequence analysis tools. Nucleic Acids Res..

[56] Quast C., Pruesse E., Yilmaz P., Gerken J., Schweer T., Yarza P., Peplies J., Glöckner F.O. (2012). The SILVA ribosomal RNA gene database project: Improved data processing and web-based tools. Nucleic Acids Res..

[57] Abarenkov K., Nilsson R.H., Larsson K.-H., Taylor A.F., May T.W., Frøslev T.G., Pawlowska J., Lindahl B., Põldmaa K., Truong C. (2024). The UNITE database for molecular identification and taxonomic communication of fungi and other eukaryotes: Sequences, taxa and classifications reconsidered. Nucleic Acids Res..

[58] Fox J., Leanage A. (2016). R and the Journal of Statistical Software. J. Stat. Softw..

