# Supplementary material for: Core and Variable Fractions of the Rhizosphere Microbiome Across Five Field-Grown Mentha Accessions
Source: Plants (Basel). 2026 Oct 9;15(20):3081. doi: 10.3390/plants15203081 (PMC13708378; doi:10.3390/plants15203081)
Supplement: Supplementary file 1 [file plants-15-03081-s001.zip › plants-4596963-Supplementary Materials.pdf]

Supplementary Materials

The accession codes used throughout the Supplementary Materials are B1 (*Mentha spicata*), B10 (*M.* × *villosa*), B12 (*M. longifolia*), B20 (*M.* × *piperita*), and J17 (*M. suaveolens*). Bulk denotes managed inter-row soil collected outside the *Mentha* accession plots. The suffixes a–c indicate the three spatial soil samples collected within each accession plot or managed inter-row soil group.

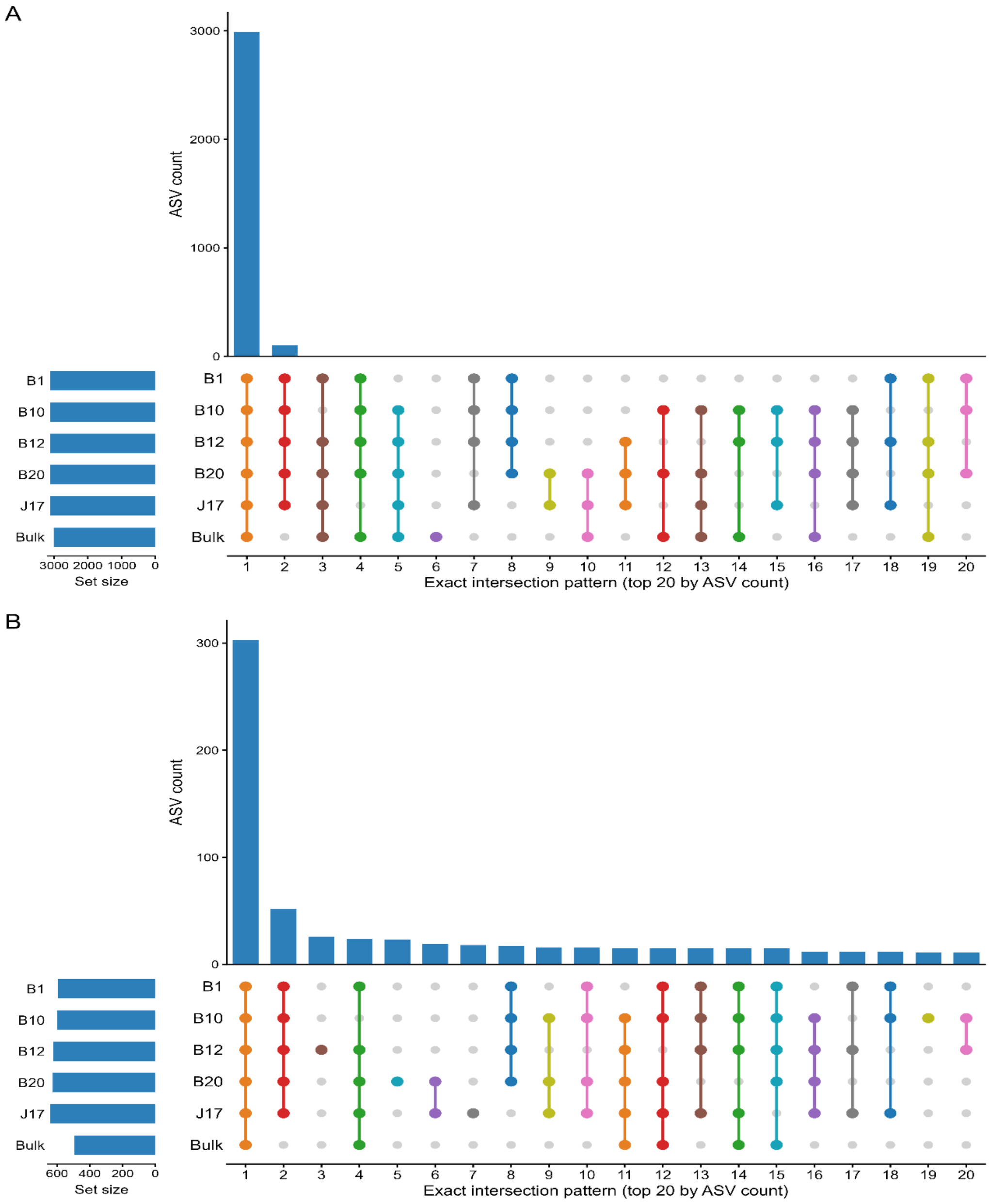

Figure S1. UpSet analysis of shared bacterial and fungal ASVs using the ≥1/3 within-group prevalence criterion. Exact intersections of bacterial 16S rRNA gene ASVs (A) and fungal ITS ASVs (B) are shown among the five *Mentha* accession plots and managed inter-row soil. An ASV was considered present in an accession plot or the managed inter-row soil group when detected in at least one of the three spatial samples. Horizontal bars indicate set sizes, and vertical bars show the 20 largest exact intersections.

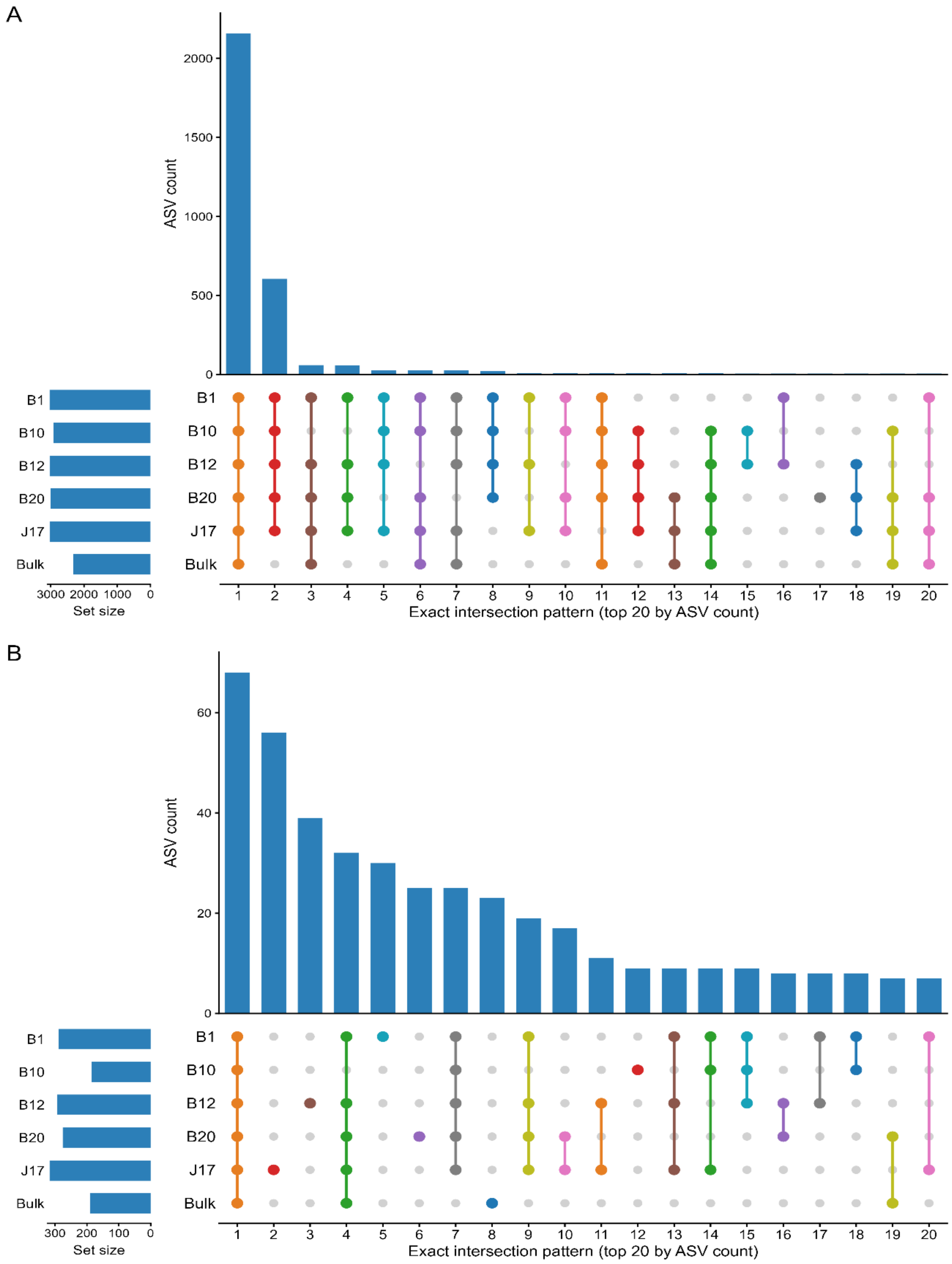

Figure S2. UpSet analysis of shared bacterial and fungal ASVs using the 3/3 within-group prevalence criterion. Exact intersections of bacterial 16S rRNA gene ASVs (A) and fungal ITS ASVs (B) are shown among the five *Mentha* accession plots and managed inter-row soil. An ASV was considered present in an accession plot or the managed inter-row soil group when detected in all three spatial samples. Horizontal bars indicate set sizes, and vertical bars show the 20 largest exact intersections.

Table S1 Essential oil composition of the analyzed *Mentha* accessions

Compound	RT[a]	LRI[b]	A. LRI[c]	B1	B10	B12	B20	J17
α-Pinene	5.56	938	932	0.39±0.18	0.72±0.01	0.45±0.26	0.39±0.16	0.17±0.25
Sabinene	6.52	976	969	0.78±0.13	0.56±0.21	0.43±0.13	0.38±0.10	0.11±0.16
β-Pinene	6.64	981	974	1.03±0.31	1.04±0.28	0.77±0.26	0.61±0.16	0.14±0.21
1-octen-3-ol	6.81	987	974	0.00±0.00	0.00±0.00	0.00±0.00	0.00±0.00	0.35±0.05
3-Octanol	6.88	990	982	0.00±0.00	0.10±0.13	0.11±0.01	0.21±0.00	0.24±0.05
β-Myrcene	6.99	995	988	1.74±0.20	1.83±0.53	0.73±0.11	0.16±0.06	0.27±0.09
α-Terpinene	7.79	1018	1014	0.06±0.08	0.00±0.00	0.00±0.00	0.00±0.00	0.00±0.00
Limonene	8.19	1029	1024	8.57±1.83	24.62±2.79	6.22±0.89	1.50±0.23	1.49±0.35
1,8-Cineole	8.38	1034	1026	8.58±0.87	5.54±0.35	1.66±0.28	4.75±0.07	0.00±0.00
(E)-Ocymene (trans-beta-ocymene)	8.85	1046	1044	0.19±0.01	0.07±0.11	0.32±0.03	0.24±0.04	0.14±0.01
γ-Terpinene	9.20	1056	1054	0.42±0.08	0.94±0.47	0.00±0.00	1.01±0.07	8.92±0.14
Trans-sabinene-hydrate	9.73	1070	1098	10.87±0.21	0.00±0.00	0.00±0.00	0.00±0.00	0.00±0.00
α-Terpinolene	10.29	1085	1086	0.15±0.06	0.00±0.00	0.00±0.00	0.00±0.00	0.00±0.00
Cis-sabinene-hydrate	10.72	1096	1065	0.17±0.24	0.00±0.00	0.00±0.00	0.00±0.00	0.00±0.00
Linalool	10.76	1097	1095	0.00±0.00	0.00±0.00	0.00±0.00	0.16±0.01	0.05±0.07
Octen-3-yl acetate	11.19	1108	1110	0.00±0.00	0.00±0.00	0.00±0.00	0.00±0.00	0.35±0.01
Menthone	13.27	1158	1148	0.00±0.00	0.00±0.00	0.23±0.04	30.20±0.42	0.59±0.63
Menthofuran	13.28	1158	1159	0.00±0.00	0.00±0.00	0.00±0.00	5.44±0.91	0.00±0.00
Isoborneol	13.43	1162	1179	0.00±0.00	0.00±0.00	0.00±0.00	0.00±0.00	0.15±0.22
α-Terpineol (cis-dehydro)	13.49	1164	1143	0.44±0.19	0.08±0.11	0.00±0.00	0.00±0.00	0.11±0.16
Isomenthone	13.67	1168	1293	0.00±0.00	0.00±0.00	0.00±0.00	6.66±0.31	0.00±0.00
Menthol	13.80	1171	1167	0.00±0.00	0.00±0.00	0.00±0.00	25.98±1.07	0.00±0.00
Terpinene-4-ol	13.96	1175	1174	1.38±0.45	0.00±0.00	0.00±0.00	0.66±0.08	0.42±0.00
Isomenthol	14.26	1182	1179	0.00±0.00	0.00±0.00	0.00±0.00	0.40±0.02	0.00±0.00
α-Terpineol	14.55	1189	1186	0.62±0.14	0.00±0.00	0.00±0.00	0.00±0.00	0.06±0.08

Dihydro-carveol	14.68	1192	1192	0.67±0.26	0.70±0.55	0.00±0.00	0.00±0.00	0.00±0.00
Cis-dihydrocarvone	14.74	1194	1191	0.79±0.12	0.46±0.65	39.98±0.53	0.00±0.00	0.00±0.00
Trans-dihydrocarvone	15.12	1203	1200	0.00±0.00	0.00±0.00	29.17±0.19	0.00±0.00	0.00±0.00
Trans-carveol	15.73	1217	1215	0.00±0.00	0.00±0.00	1.24±0.38	0.00±0.00	0.00±0.00
Hexenyl 2-methyl butanoat (3Z)	16.20	1228	1229	0.00±0.00	0.00±0.00	0.00±0.00	0.00±0.00	0.07±0.10
Pulegon	16.50	1236	1233	0.00±0.00	0.00±0.00	0.00±0.00	5.41±0.35	0.05±0.07
L-carvone	16.71	1241	1239	54.98±2.03	61.75±2.18	0.58±0.13	0.00±0.00	0.00±0.00
Piperitone	17.08	1249	1249	0.00±0.00	0.00±0.00	0.00±0.00	1.83±0.21	0.00±0.00
Cis-piperitone-epoxide	17.42	1257	1250	0.00±0.00	0.00±0.00	0.00±0.00	0.00±0.00	64.43±0.31
Thymol	18.81	1290	1289	0.00±0.00	0.00±0.00	0.07±0.10	0.00±0.00	0.64±0.42
Menthyl-acetate	18.84	1291	1294	0.00±0.00	0.00±0.00	0.00±0.00	4.49±0.24	0.00±0.00
Isomenthyl-acetate	19.04	1296	1304	0.00±0.00	0.00±0.00	0.00±0.00	0.15±0.01	0.00±0.00
Dihydro-carveol-acetate (iso)	20.11	1324	1312	0.31±0.18	0.00±0.00	2.09±0.09	0.00±0.00	0.00±0.00
Dihydro-carveol-acetate (neoiso)	21.57	1364	1356	0.00±0.00	0.00±0.00	1.27±0.08	0.00±0.00	0.00±0.00
Piperitenone oxide	21.97	1375	1366	0.00±0.00	0.00±0.00	0.00±0.00	0.00±0.00	8.02±0.33
β-Bourbonene	22.26	1383	1387	1.32±0.30	0.28±0.39	0.36±0.02	0.41±0.03	0.14±0.04
β-Elemene	22.55	1391	1389	0.39±0.17	0.00±0.00	0.14±0.02	0.77±0.02	0.06±0.08
Caryophyllene (4,8-α-epoxy)	23.62	1418	1415	0.00±0.00	0.00±0.00	0.00±0.00	0.00±0.00	1.62±0.01
β-Caryophyllene	23.68	1420	1417	1.75±0.37	0.19±0.27	5.01±0.03	3.55±0.20	3.71±0.08
α-Humulene	25.07	1454	1452	0.00±0.00	0.00±0.00	0.11±0.01	0.00±0.00	0.26±0.02
β-farnesene	25.27	1459	1454	0.00±0.00	0.00±0.00	0.00±0.00	0.11±0.00	0.00±0.00
Germacrene-D	26.18	1482	1480	0.83±0.37	0.47±0.67	5.88±0.07	3.13±0.18	4.46±0.09
Bicyclogermacrene	26.81	1497	1500	0.29±0.18	0.08±0.11	0.91±0.06	0.30±0.02	0.00±0.00
Germacrene-A	27.06	1504	1508	0.00±0.00	0.00±0.00	0.00±0.00	0.10±0.00	0.00±0.00
δ-Cadinene	27.80	1524	1513	0.17±0.09	0.00±0.00	0.22±0.03	0.00±0.00	0.00±0.00
β-Cadinene	29.87	1581	1537	0.00±0.00	0.00±0.00	0.15±0.00	0.00±0.00	0.00±0.00
Viridiflorol	30.49	1598	1592	0.00±0.00	0.00±0.00	0.21±0.30	0.14±0.02	0.00±0.00

**Sum:**				**97.50±1.63**	**99.60±0.56**	**98.75±0.00**	**99.12±0.16**	**97.05±0.50**

Values (mean % ± standard deviation, *n* = 3 per accession) represent the relative percentage of each compound within the total oil.
[a]RT: Retention Time; [b]LRI: Linear Retention Index; [c]A. LRI: Adams Linear Retention Index

Table S2. Summary of bacterial 16S rRNA gene sequencing data and taxonomic richness across *Mentha* rhizosphere and managed inter-row soil samples

Samples	Nb domain	Nb phylum	Nb class	Nb order	Nb family	Nb genus	Nb species	Nb ASV	Nb sequences
B1a	1	27	62	131	199	418	515	3,082	122,671
B1b	1	27	62	131	201	421	517	3,070	137,669
B1c	1	27	62	131	201	424	519	3,090	130,790
B10a	1	27	62	129	197	409	497	2,996	140,816
B10b	1	27	62	131	201	422	518	3,084	129,284
B10c	1	27	62	128	197	414	509	3,030	130,281
B12a	1	27	62	131	202	424	521	3,082	134,985
B12b	1	27	62	132	202	424	519	3,084	133,714
B12c	1	27	62	132	202	425	517	3,078	141,470
B20a	1	27	62	132	201	422	518	3,060	114,039
B20b	1	27	62	131	201	423	519	3,079	153,326
B20c	1	27	62	132	202	429	526	3,092	132,046
J17a	1	27	62	132	202	424	519	3,082	125,045
J17b	1	27	62	131	202	425	517	3,066	121,293
J17c	1	27	62	132	202	426	523	3,080	112,125
Bulk_a	1	24	59	123	187	379	460	2,590	147,693
Bulk_b	1	26	61	126	193	396	478	2,788	136,155
Bulk_c	1	26	61	127	194	395	474	2,768	167,262
**Total**	**1**	**27**	**62**	**132**	**203**	**433**	**529**	**3,128**	**2,410,664**

All sample-level entries are absolute counts for individual samples. "Total" indicates the combined values across all samples. For sequence reads, values represent the sum across samples; for taxonomic groups and ASVs, values represent the total number detected in the full dataset.

The suffixes a–c denote the three within-plot subsamples collected from each accession plot, whereas Bulk_a–Bulk_c denote the three individual managed inter-row soil samples.

Sequence-processing summary. The bacterial 16S dataset contained 3,642,821 input sequences at the FROGS preprocessing step. After paired-end assembly, primer and length filtering, and removal of sequences containing ambiguous bases, 3,326,459 sequences were retained. SWARM clustering generated 209,924 initial sequence clusters. Chimera removal retained 112,263 clusters representing 3,210,877 sequences. Subsequent abundance filtering (<0.005% of the total sequence count) retained 3,130 sequence clusters (ASVs) representing 2,416,128 sequences. Following taxonomic affiliation, one chloroplast-affiliated ASV and one mitochondrial-affiliated ASV were removed as non-target sequences, resulting in the final dataset of 3,128 ASVs and 2,410,664 sequences reported in Table S2.

Taxonomic-label handling. Sequence clusters that could not be resolved at a lower taxonomic rank were retained in the dataset. For downstream taxonomic summaries, unresolved assignments were relabeled according to the lowest reliably identified higher rank using the format unclassified_[taxon] (for example, an unresolved genus assigned to Vicinamibacterales was labeled "unclassified_Vicinamibacterales"). This relabeling affected taxonomic nomenclature only and did not alter ASV or sequence counts.

Relative abundance of a given ASV or taxon within a sample was calculated as:

relative abundance (%) = (number of reads assigned to the ASV or taxon / total number of retained reads in that sample) × 100.

For higher taxonomic ranks, reads from all ASVs assigned to the same taxon were summed before calculation. Group-level values shown in figures or summaries represent the arithmetic mean of the three individual sample-level relative abundances within each accession plot or managed inter-row soil group.

Table S3. Summary of fungal ITS gene sequencing data and taxonomic richness across *Mentha* rhizosphere and managed inter-row soil samples

Samples	Nb domain	Nb phylum	Nb class	Nb order	Nb family	Nb genus	Nb species	Nb ASV	Nb sequences
B1a	1	10	28	54	102	175	232	445	91,346
B1b	1	10	26	52	99	166	225	466	75,962
B1c	1	10	30	53	96	157	209	414	109,834
B10a	1	8	19	39	74	121	165	296	110,829
B10b	1	11	30	58	110	191	262	508	109,944
B10c	1	10	21	38	74	137	178	327	113,608
B12a	1	10	29	52	98	166	219	442	117,606
B12b	1	11	34	60	104	177	238	474	117,935
B12c	1	11	30	59	104	173	228	440	124,903
B20a	1	11	34	60	108	185	242	470	116,044
B20b	1	9	27	51	96	157	206	402	127,783
B20c	1	10	30	55	103	185	245	460	92,226
J17a	1	12	33	63	115	191	253	485	75,556
J17b	1	11	29	56	103	180	247	457	76,124
J17c	1	11	31	59	109	192	254	484	82,151
Bulk_a	1	11	27	49	90	150	198	393	93,298
Bulk_b	1	6	15	31	59	99	126	238	122,735
Bulk_c	1	11	31	55	98	157	203	410	124,699
**Total**	**1**	**12**	**39**	**79**	**153**	**281**	**388**	**859**	**1,882,583**

All sample-level entries are absolute counts for individual samples. "Total" indicates the combined values across all samples. For sequence reads, values represent the sum across samples; for taxonomic groups and ASVs, values represent the total number detected in the full dataset.

The suffixes a–c denote the three within-plot subsamples collected from each accession plot, whereas Bulk_a–Bulk_c denote the three individual managed inter-row soil samples.

Sequence-processing summary. The fungal ITS dataset contained 2,896,500 input sequences at the FROGS preprocessing step. After paired-end assembly, expected-length filtering, and removal of sequences containing ambiguous bases, 2,283,620 sequences were retained. SWARM clustering generated 297,440 initial sequence clusters. Chimera removal retained 272,568 clusters representing 2,250,016 sequences. Subsequent abundance filtering (<0.005% of the total sequence count) retained 863 sequence clusters (ASVs) representing 1,888,387 sequences. ITSx filtering retained 862 ASVs representing 1,888,256 sequences. Following taxonomic affiliation, three non-fungal ASVs were removed, resulting in the final dataset of 859 ASVs and 1,882,583 sequences reported in Table S3.

Taxonomic-label handling and relative-abundance calculations were performed as described above for Table S2.

Thirty-eight ASVs could be assigned to Fungi but could not be resolved at phylum level; these were retained as "unclassified_Fungi" and represented 12,387 sequences (0.66% of the final fungal dataset).

Table S4. Alpha-diversity indices of bacterial communities across the five *Mentha* accession plots and managed inter-row soil

Accession	Observed	Chao1	Shannon	InvSimpson
B1	3072.00 ± 13.11a	3091.49 ± 11.96a	7.303 ± 0.016a	711.87 ± 24.72a
B10	3024.67 ± 51.50a	3060.63 ± 39.24a	7.212 ± 0.057a	639.68 ± 54.88a
B12	3071.00 ± 5.29a	3104.97 ± 20.44a	7.275 ± 0.022a	706.29 ± 22.11a
B20	3070.00 ± 13.11a	3093.16 ± 17.53a	7.257 ± 0.018a	599.45 ± 80.70a
J17	3074.67 ± 8.39a	3089.87 ± 7.71a	7.276 ± 0.022a	676.17 ± 40.55a
bulk	2676.67 ± 105.99b	2761.61 ± 111.57b	6.864 ± 0.109b	375.89 ± 47.39b
Global p	$p < 0.001$	$p < 0.001$	$p < 0.001$	$p < 0.001$

Values represent means ± standard deviation ($n = 3$). Different lowercase letters within a column indicate significant differences among groups according to one-way ANOVA followed by Tukey's HSD test ($p < 0.05$). Global p values correspond to the one-way ANOVA.

Table S5. Alpha-diversity indices of fungal communities across the five *Mentha* accession plots and managed inter-row soil

Accession	Observed	Chao1	Shannon	InvSimpson
B1	439.67 ± 28.75	457.74 ± 32.15	4.553 ± 0.161ab	44.30 ± 9.67
B10	363.67 ± 114.72	422.88 ± 94.94	4.255 ± 0.225bc	35.01 ± 12.17
B12	437.00 ± 14.80	468.11 ± 26.63	4.687 ± 0.209a	56.19 ± 17.98
B20	437.67 ± 39.63	446.32 ± 40.55	4.348 ± 0.053abc	35.36 ± 3.11
J17	474.67 ± 15.37	490.66 ± 14.34	4.573 ± 0.070ab	43.32 ± 5.23
bulk	339.33 ± 99.30	367.17 ± 69.80	4.146 ± 0.057c	28.16 ± 3.61
Global p	$p = 0.171$	$p = 0.166$	$p = 0.005$	$p = 0.067$

Values represent means ± standard deviation ($n = 3$). Different lowercase letters within a column indicate significant differences among groups according to one-way ANOVA followed by Tukey's HSD test ($p < 0.05$). Global p values correspond to the one-way ANOVA.

Table S6. Sensitivity analysis of core and shared bacterial and fungal ASV fractions before and after rarefaction

Category	16S non-rarefied ASVs *n* (%)	16S non-rarefied cumulative abundance (%)	16S rarefied ASVs *n* (%)	16S rarefied cumulative abundance (%)	ITS non-rarefied ASVs *n* (%)	ITS non-rarefied cumulative abundance (%)	ITS rarefied ASVs *n* (%)	ITS rarefied cumulative abundance (%)
Total filtered ASVs	3,128 (100)	–	3,128 (100)	–	859 (100)	–	859 (100)	–
Universal core, 18/18	2,158 (68.99)	86.04 ± 1.88	2,081 (66.53)	85.00 ± 1.99	68 (7.92)	51.29 ± 8.65	61 (7.10)	48.64 ± 8.13
Strict rhizosphere core, 15/15	2,762 (88.30)	96.00 ± 1.04	2,714 (86.76)	95.52 ± 1.08	93 (10.83)	58.80 ± 9.48	89 (10.36)	58.48 ± 9.35
Rhizosphere core, ≥12/15	3,062 (97.89)	99.33 ± 0.40	3,047 (97.41)	99.21 ± 0.43	243 (28.29)	78.47 ± 5.72	231 (26.89)	77.56 ± 6.01
Shared fraction across all five *Mentha* accession plots, ≥2/3 each	3,005 (96.07)	98.65 ± 0.52	2,984 (95.40)	98.40 ± 0.59	188 (21.89)	72.83 ± 7.66	177 (20.61)	72.36 ± 7.48
Rhizosphere-associated, ≥12/15 and absent from bulk	91 (2.91)	1.46 ± 0.54	101 (3.23)	1.66 ± 0.57	13 (1.51)	2.59 ± 5.45	14 (1.63)	2.72 ± 5.39
Accession-plot-associated exclusive	0 (0)	0	0 (0)	0	29 (3.38)	0.79 ± 0.67	26 (3.03)	0.72 ± 0.58

ASV counts are given as absolute numbers (*n*), with percentages of the total filtered ASV pool in parentheses. Cumulative abundance represents the summed relative abundance of ASVs assigned to each category within individual rhizosphere samples and is reported as mean ± SD across the 15 rhizosphere samples. The primary analysis was based on the filtered, non-rarefied abundance tables. For the sensitivity analysis, the same definitions and prevalence criteria were applied after rarefaction to 112,125 sequences per sample for the bacterial dataset and 75,556 sequences per sample for the fungal dataset.

Table S7. Fixed-rank sensitivity analysis of shared bacterial and fungal community fractions and the effect of unresolved taxonomic assignments

**A. Taxonomic assignment completeness at genus and family ranks**						
**Dataset**	**Rank**	**Total ASVs**	**Resolved ASVs, *n* (%)**	**Unresolved ASVs, *n* (%)**	**Resolved rhizosphere reads, *n* (%)**	**Unresolved rhizosphere reads, *n* (%)**
16S	Genus	3,128	1,446 (46.23)	1,682 (53.77)	914,015 (46.64)	1,045,539 (53.36)
16S	Family	3,128	2,364 (75.58)	764 (24.42)	1,458,964 (74.45)	500,590 (25.55)
ITS	Genus	859	638 (74.27)	221 (25.73)	1,289,225 (83.62)	252,626 (16.38)
ITS	Family	859	716 (83.35)	143 (16.65)	1,442,601 (93.56)	99,250 (6.44)
**B. Shared-community fractions after aggregation at fixed taxonomic ranks**						
**Taxonomic level and treatment**	**16S total units, *n***	**16S shared, *n* (%)**	**16S cumulative abundance (%)**	**ITS total units, *n***	**ITS shared, *n* (%)**	**ITS cumulative abundance (%)**
ASV, primary analysis	3,128	3,005 (96.07)	98.65 ± 0.52	859	188 (21.89)	72.83 ± 7.66
Genus, resolved taxa only	293	275 (93.86)	99.42 ± 0.38*	234	75 (32.05)	84.85 ± 7.38*
Genus-column aggregation, all labels retained	433	413 (95.38)	99.72 ± 0.18	281	89 (31.67)	85.00 ± 8.57
Family, resolved taxa only	143	140 (97.90)	99.91 ± 0.11*	130	48 (36.92)	92.65 ± 3.93*
Family-column aggregation, all labels retained	203	199 (98.03)	99.93 ± 0.08	153	57 (37.25)	92.22 ± 4.13

Analyses were performed using the filtered, non-rarefied abundance tables and the same primary within-plot prevalence criterion used for the ASV-level shared-community analysis. A taxonomic unit was considered present within an accession plot when it was

detected in at least two of the three spatial samples, and a unit was classified as shared when this criterion was fulfilled in all five *Mentha* accession plots.

In Panel A, “resolved” denotes sequence clusters assigned to the target taxonomic rank, whereas “unresolved” denotes clusters for which the target rank could not be assigned and the corresponding taxonomy field therefore contained an “unclassified_” label referring to the lowest reliably identified higher taxonomic level. Percentages of rhizosphere reads were calculated across the 15 *Mentha* rhizosphere samples; the bacterial and fungal rhizosphere datasets contained 1,959,554 and 1,541,851 reads, respectively. The higher proportion of unresolved assignments in the bacterial dataset, particularly at genus level, means that exclusion of unresolved lineages results in substantially greater loss of 16S than ITS sequence information.

In the “resolved taxa only” analysis in Panel B, clusters lacking assignment to the specified rank were excluded before aggregation, so that only genuine genus- or family-level assignments were compared. The total-unit count therefore represents the number of distinct resolved genera or families detected in the complete dataset. Cumulative abundance values marked with an asterisk were calculated within the retained, rank-resolved read pool of each individual rhizosphere sample and are therefore not directly equivalent to percentages calculated from the complete read pool.

In the “all labels retained” analysis, no sequence clusters were discarded. Clusters with a resolved genus or family assignment were aggregated by that taxon, whereas unresolved clusters sharing the same existing unclassified_ taxonomic label were combined into a single operational bin. These unresolved bins should not be interpreted as formally identified genera or families, because multiple distinct lower-level taxa may be represented within the same unresolved higher-level lineage. Conversely, artificially separating such bins into multiple putative genera or families was avoided because the available marker data do not support that distinction. Cumulative abundances for the ASV-level and all-label analyses were calculated relative to the complete read count of each rhizosphere sample and are reported as mean ± SD across the 15 samples.

The resolved-only and all-label analyses therefore represent complementary sensitivity analyses. The former provides a strict fixed-rank comparison at the cost of excluding unresolved sequence clusters, whereas the latter retains the complete sequence dataset but treats unresolved higher-level assignments as operational taxonomic bins. The similar shared-community proportions obtained with the two approaches indicate that the bacterial–fungal contrast was not driven primarily by the handling of unresolved taxonomic assignments. The ASV-level values are included for reference and correspond to the primary analysis reported in Table 2.

Table S8. Representative taxa within core, shared, rhizosphere-associated, and accession-plot-associated exclusive ASV fractions

ASV fraction	Community / accession	No. of ASVs	Representative taxa
Strict rhizosphere core (15/15)	Bacteria	2762	unclassified_Vicinamibacterales; unclassified_Gaiellales; *Vicinamibacteraceae*; *Nocardioidaceae*; 67-14; *Gaiella*; *Nocardioides*; *Rubrobacter*
Rhizosphere core (≥12/15)	Bacteria	3062	unclassified_Vicinamibacterales; unclassified_Gaiellales; *Vicinamibacteraceae*; *Nocardioidaceae*; *Xanthobacteraceae*; *Sphingomonadaceae*; *Gaiella*; *Nocardioides*
Shared fraction across five accession plots (≥2/3 each)	Bacteria	3005	unclassified_Vicinamibacterales; unclassified_Gaiellales; *Vicinamibacteraceae*; *Nocardioidaceae*; 67-14; *Gaiella*; *Nocardioides*; *Rubrobacter*
Rhizosphere-associated (≥12/15; managed inter-row soil absent)	Bacteria	91	*Chthoniobacteraceae*; *Pedosphaeraceae*; *Microscillaceae*; *Chitinophagaceae*; *Sphingomonadaceae*; *Chthoniobacter*; *Novosphingobium*; *Williamsia*; *Chryseolinea*
Strict rhizosphere core (15/15)	Fungi	93	*Nectriaceae*; *Didymellaceae*; *Chaetomiaceae*; *Plectosphaerellaceae*; *Stachybotryaceae*; *Mortierellaceae*; *Fusarium*; *Humicola*; *Plectosphaerella*; *Didymella*
Rhizosphere core (≥12/15)	Fungi	243	*Nectriaceae*; *Didymellaceae*; *Chaetomiaceae*; *Plectosphaerellaceae*; *Stachybotryaceae*; *Mortierellaceae*; *Fusarium*; *Humicola*; *Plectosphaerella*; *Didymella*
Shared fraction across five accession plots (≥2/3 each)	Fungi	188	*Nectriaceae*; *Didymellaceae*; *Chaetomiaceae*; *Mortierellaceae*; *Plectosphaerellaceae*; *Stachybotryaceae*; *Fusarium*; *Humicola*; *Plectosphaerella*; *Didymella*
Rhizosphere-associated (≥12/15; managed inter-row soil absent)	Fungi	13	*Plectosphaerellaceae*; *Bionectriaceae*; Hypocreales-affiliated taxa; *Paragibellulopsis*; *Nectriella*; *Fusariella*; *Vishniacozyma*; *Beauveria*

Accession-plot-associated exclusive	Bacteria	0	None detected
Accession-plot-associated exclusive (Fungi)	*M. spicata* B1	3	*Oliveonia*; *Dominikia gansuensis*; *Operculomyces laminatus*
Accession-plot-associated exclusive (Fungi)	*M.* × *villosa* B10	3	*Preussia*; *Tulosesus pallidus*; one unresolved fungal ASV
Accession-plot-associated exclusive (Fungi)	*M. longifolia* B12	11	Rozellomycota-affiliated ASVs; *Coprinopsis* sp.; Ceratobasidiaceae; *Diversispora eburnea*
Accession-plot-associated exclusive (Fungi)	*M.* × *piperita* B20	7	*Rhizophlyctis* sp.; *Typhula*; *Pezizaceae*-affiliated ASV; *Ramophialophora petraea*; *Scutellinia* sp.
Accession-plot-associated exclusive (Fungi)	*M. suaveolens* J17	5	*Ochronectria thailandica*; *Acrocalymma medicaginis*; Helotiales-affiliated ASV; *Psathyrella panaeoloides*

Representative taxa were selected from the most abundant and/or most taxonomically resolved ASVs within each category. Core and rhizosphere-associated categories are not mutually exclusive. Accession-plot-associated exclusive ASVs were defined by presence in at least two of three samples from one accession plot and absence from all other *Mentha* and managed inter-row soil samples. The listed taxa are descriptive and should not be interpreted as accession-specific biomarkers.
